# Association between maternal depression during pregnancy and newborn DNA methylation

**DOI:** 10.1038/s41398-021-01697-w

**Published:** 2021-11-08

**Authors:** Emily Drzymalla, Nicole Gladish, Nastassja Koen, Michael P. Epstein, Michael S. Kobor, Heather J. Zar, Dan J. Stein, Anke Hüls

**Affiliations:** 1 Department of Epidemiology, Rollins School of Public Health, Emory University, Atlanta, GA USA; 2 Department of Medical Genetics, University of British Columbia, Vancouver, BC Canada; 3 BC Children’s Hospital Research Institute, Vancouver, BC Canada; 4 Centre for Molecular Medicine and Therapeutics, Vancouver, BC Canada; 5 Neuroscience Institute, University of Cape Town, Cape Town, South Africa; 6 Department of Psychiatry and Mental Health, University of Cape Town, Cape Town, South Africa; 7 South African Medical Research Council (SAMRC) Unit on Risk and Resilience in Mental Disorders, University of Cape Town, Cape Town, South Africa; 8 Department of Human Genetics, School of Medicine, Emory University, Atlanta, GA USA; 9 Department of Paediatrics and Child Health, Red Cross War Memorial Children’s Hospital, University of Cape Town, Cape Town, South Africa; 10 South African Medical Research Council (SAMRC) Unit on Child & Adolescent Health, Cape Town, South Africa; 11 Gangarosa Department of Environmental Health, Rollins School of Public Health, Emory University, Atlanta, GA USA

**Keywords:** Genetics, Depression

## Abstract

Around 15–65% of women globally experience depression during pregnancy, prevalence being particularly high in low- and middle-income countries. Prenatal depression has been associated with adverse birth and child development outcomes. DNA methylation (DNAm) may aid in understanding this association. In this project, we analyzed associations between prenatal depression and DNAm from cord blood from participants of the South African Drakenstein Child Health Study. We examined DNAm in an epigenome-wide association study (EWAS) of 248 mother-child pairs. DNAm was measured using the Infinium MethylationEPIC (*N* = 145) and the Infinium HumanMethylation450 (*N* = 103) arrays. Prenatal depression scores, obtained with the Edinburgh Postnatal Depression Scale (EPDS) and the Beck Depression Inventory-II (BDI-II), were analyzed as continuous and dichotomized variables. We used linear robust models to estimate associations between depression and newborn DNAm, adjusted for measured (smoking status, household income, sex, preterm birth, cell type proportions, and genetic principal components) and unmeasured confounding using Cate and Bacon algorithms. Bonferroni correction was used to adjust for multiple testing. DMRcate and dmrff were used to test for differentially methylated regions (DMRs). Differential DNAm was significantly associated with BDI-II variables, in cg16473797 (Δ beta = −1.10E-02, *p* = 6.87E-08), cg23262030 (Δ beta per BDI-II total IQR = 1.47E-03, *p* = 1.18E-07), and cg04859497 (Δ beta = −6.42E-02, *p* = 1.06E-09). Five DMRs were associated with at least two depression variables. Further studies are needed to replicate these findings and investigate their biological impact.

## Introduction

Prenatal depression affects about 15–65% of women around the world with a higher percentage in low to middle-income countries (LMICs) than high-income countries (HICs) [1]. Adverse birth and child development outcomes, such as low birth weight, pre-term birth, and developmental delay, have been observed in children whose mothers experienced prenatal depression [1, 2]. Epigenetics has been hypothesized to play a role in this association. Prenatal development is a crucial and vulnerable period for the epigenome due to epigenetic reprogramming that occurs for both DNA methylation (DNAm) and histone modifications during this time [3]. With the exception of a few regions such as imprinted genes, the epigenome is reprogrammed by the global decrease in DNAm pre-implantation and then increase in DNAm following implantation for processes such as organogenesis [4]. Prenatal exposures such as tobacco smoke [5], maternal stress [6], or toxins [7] can affect the child’s epigenome during prenatal development. Changes in the infant’s epigenetic mechanisms, such as DNAm, as a result of prenatal depression, may provide insight into this association either as a biomarker or as a possible mediating factor in biological pathways.

Previous studies investigating the association between prenatal depression and differential DNAm have focused on candidate genes such as *NR3C1* and *SLC6A4* [8, 9]. Children exposed to prenatal depression have been shown to have increased DNAm in *NR3C1* and decreased DNAm in *SLC6A4* [8, 9]. Epigenome-wide association studies (EWAS) have also investigated associations between prenatal depression and differential DNAm [10, 11]. Two of these studies found a combined total of five CpG sites (cg08667740, cg22868225, cg06808585, cg05245515, and cg15264806) and 39 differentially methylated regions (DMRs) associated with prenatal depression [10, 11]. These previous studies included mother-child pairs only from high-income countries including Norway, the Netherlands, the United Kingdom, and the United States [10–12].

As women from LMICs are particularly vulnerable to prenatal depression [1], this study aimed to investigate this association using the Drakenstein child health study (DCHS), a population-based birth cohort in South Africa [13, 14]. This cohort is representative of several aspects of the LMIC context and allows for the study of potential associations between prenatal depression and DNAm in this setting [13].

## Materials and methods

### Study population

The study population consisted of 248 mother-child pairs from the DCHS with data available for the Edinburgh Postnatal Depression Scale (EPDS) and the Beck Depression Inventory-II (BDI-II) scores, cord blood DNAm, and covariates. Participants were recruited between March 2012 and March 2015 from two primary care clinics, TC Newman or Mbekweni [13, 15]. Mothers were enrolled during their second trimester and followed until the child was at least five years old [13–16].

Ethical approval was given from the Human Research Ethics Committee of the Faculty of Health Sciences of the University of Cape Town for human subjects’ research and written consent was obtained from the mothers [13, 15].

### DNA methylation measurements

DNA methylation was measured from cord blood collected at delivery by either the MethylationEPIC BeadChips (EPIC; *n* = 145) or the Illumina Infinium HumanMethylation450 BeadChips (450K; *n* = 103) [13, 15]. The subgroup that was selected for the second set of DNAm analyses (EPIC, *n* = 145) was enriched for maternal trauma exposure/post-traumatic stress disorder (PTSD).

Pre-processing and statistics were done using R 3.5.1. Raw iDat files were imported to RStudio where intensity values were converted into beta values. The 450K array had 426,378 probes while the EPIC array contained 781,536 probes. Pre-processing was performed in each array separately but with identical pre-processing steps. Background subtraction, colour correction and normalization were performed using the preprocessFunnorm function. After sample and probe filtering, 120 samples and 426,378 probes remained for the 450K dataset with 153 samples and 781,536 probes with the EPIC dataset. Batch effects were removed using ComBat from the R package sva. Cord blood cell type composition was predicted using the most recent cord blood reference data set and the IDOL algorithm and probe selection.

### Depression measurements

Prenatal depression was assessed with both the EPDS and BDI-II administered at 28–32 weeks gestation [13, 15]. The EPDS scale has 10 questions, scores ranging from 0 to 30, and was designed to screen for postnatal depression [17]. This scale has been verified for prenatal depression in an African setting [18]. The BDI-II scale has 21 questions, scores ranging from 0 to 63, and is used to screen for depression [19]. The BDI-II scale has been validated for prenatal depression [20] and used for prenatal depression in countries such as Ethiopia and Kenya [21, 22]. For EPDS, thresholds of 10 and 13 are commonly used to screen for depression with 10 having a higher sensitivity and 13 having a higher specificity [23]. For BDI-II, the lower threshold of 14 is the threshold for mild depression, and a higher threshold of 20 is the threshold for moderate depression [19].

### Statistical analysis

The association between prenatal maternal depression and newborn differential methylation at individual CpG sites and DMRs were assessed in epigenome-wide association studies (EWAS). We conducted EWAS for the 450K and EPIC data separately, followed by a meta-analysis to combine the results of the CpG sites that were measured with both arrays. For each of the EWAS analyses, a multivariable robust linear regression model with empirical Bayes using the limma R package was fitted [24]. The dependent variable was cord blood DNAm, with depression variables as the independent variable while adjusting for the following covariates: mother’s smoking status, household income, sex of the child, gestational age at birth, first three cell type principal components (PCs) which explained 90% of heterogeneity due to cell type [25], and first five genotype PCs for population stratification. The meta-analysis was performed imputing the individual 450K and EPIC EWAS results into METAL and running a fixed-effect model with inverse variance weighting [26]. A sensitivity analysis was performed to determine the effect of including HIV exposure as a potential confounder in the model. P-values were additionally adjusted for bias and unmeasured confounding using the Bacon and Cate R packages respectively [27] (Table S1). The continuous depression scale was used as primary outcomes, followed by analyses of the dichotomized variables (screening for depression) as secondary outcomes. To account for multiple testing, the Bonferroni threshold was used for statistical significance (EPIC: 0.05 / 781536 CpGs = 6.40 × 10^−8^, 450K: 0.05/426378 CpGs = 1.17 × 10^−7^, meta-analysis: 0.05/386685 CpGs = 1.29 × 10^−7^). Fine-mapping of our epigenome-wide associations was done with the R package comet, that displays the region surrounding any significant CpG sites [28]. DMRs were assessed from the meta-analysis of the overlapping CpG sites from EPIC and 450K using the R package DMRcate (version 1.20.0) [29]. The input files for these analyses included the regression coefficients, standard deviations, and *p*-values from the meta-analysis of single-CpG analyses. The R package dmrff [30] was also used to evaluate DMRs by meta-analysis of the EPIC and 450K arrays. DMRs for both the DMRcate and dmrff analysis methods were defined by requiring at least two CpG sites within 1,000 bps apart and the region having a Bonferroni corrected *p*-value < 0.05. Furthermore, we used the robustness of DMRs across different depression scales as an additional validation criterion.

For any significant CpG sites or DMRs, we looked up the correlations between blood and brain DNA methylation using the public data source IMAGE-CpG, that is based on blood, saliva, buccal, and live brain tissue samples from 27 patients with medically intractable epilepsy undergoing brain resection [31].

## Results

### Study population characteristics

The analysis sample included 248 mother-child pairs with complete information for depression scores, cord blood DNAm, and relevant covariates (Table 1). DNAm was measured in the cord blood of 145 infants (58%) using the EPIC array and in 103 infants (42%) using the 450K array. Overall, 44% of the infants were female and the mean gestational age at birth was 38.75 weeks. About 21% of the mothers were smokers during pregnancy with a higher proportion of smokers in 450K data than in the EPIC data. The average EPDS score was 10.52 (sd = 5.09) with 56% defined as depressed according to the threshold of 10 and 31% according to the threshold of 13. The average BDI-II score among mothers was 13.19 (sd = 11.17) with 44% and 25% defined as depressed according to the thresholds of 14 or 20, respectively. The women in the 450K array group (*n* = 103) tended to have higher depression scores (EPDS and BDI-II) than in the EPIC data.

**Table 1:** Population characteristics for the total population and stratified by arrays.

Characteristic	Combined (*n* = 248)	EPIC (*n* = 145)	450K (*n* = 103)
Female, *n* (%)	110 (44.35%)	67 (46.21%)	43 (41.75%)
Gestational age at birth, mean (sd)	38.75 (2.19)	38.77 (2.47)	38.73 (1.73)
Household Income			
<R1000/month, *n* (%)	98 (39.52%)	58 (40.00%)	40 (38.83%)
R1000–R5000/month, *n* (%)	107 (43.15%)	64 (44.83%)	43 (41.75%)
>R5000/month, *n* (%)	43 (17.33%)	23 (15.17%)	20 (19.42%)
Maternal Smoking, *n* (%)	53 (21.37%)	27 (18.62%)	27 (26.21%)
EPIC Array, *n* (%)	145 (58.47%)	145 (100.00%)	0 (0.00%)
EPDS Continuous, mean (sd)	10.52 (5.09)	10.30 (4.64)	10.83 (5.68)
EPDS Threshold 10, *n* (%)	140 (56.45%)	83 (57.24%)	58 (56.31%)
EPDS Threshold 13, *n* (%)	78 (31.45%)	40 (27.59%)	38 (36.89%)
BDI-II Continuous, mean (sd)	13.19 (11.17)	11.26 (10.53)	15.89 (11.55)
BDI-II Threshold 14, *n* (%)	108 (43.55%)	54 (37.24%)	54 (52.43%)
BDI-II Threshold 20, *n* (%)	62 (25.00%)	26 (17.93%)	36 (34.95%)

### Primary outcomes: Maternal depression scores and newborn DNAm

**Fig. 1:**
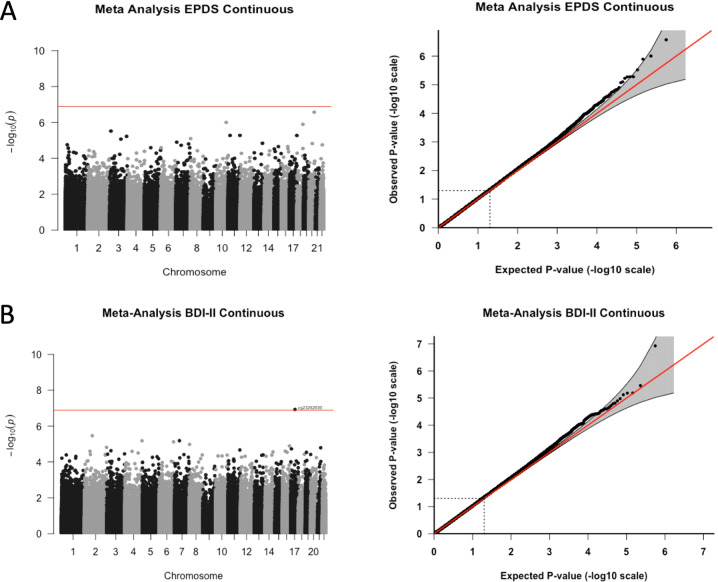
Manhattan and QQ plots for the meta-analysis of EWAS of maternal depression (primary outcomes, **A**. EPDS and **B**. BDI-II). Adjusted for covariates: mother’s smoking status, average household income, child’s sex, gestational age at birth, first three cell type PCs, and first five genotype PCs. Unmeasured confounding and bias were adjusted with Cate and Bacon R packages. Bonferroni threshold: meta-analysis = 1.29 × 10^−7^.

**Fig. 2:**
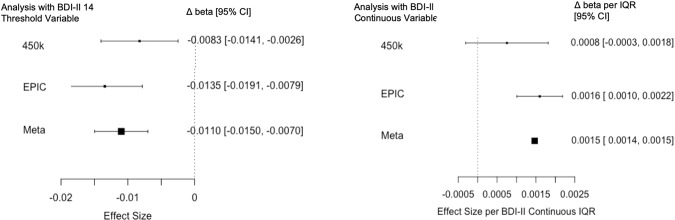
Forest plot indicating effect sizes and 95% confidence for both EWAS analyzes (450K alone and EPIC alone) and the meta-analysis for thesignificant sites. Adjusted for covariates: mother’s smoking status, average household income, child’s sex, gestational age at birth, first three cell type PCs, and first five genotype PCs. Unmeasured confounding and bias were adjusted with Cate and Bacon R packages. **A** For cg16473797 using the BDI-II 14 threshold. **B** For cg23262030 using the BDI-II continuous variable.

After accounting for bias and measured and unmeasured confounding, the EWAS of the individual 450K and EPIC data did not produce significant CpG sites for either of the continuous depression variables (EPDS and BDI-II) (Figure S1–S4). After combining the overlapping CpG sites from both arrays in a meta-analysis, we found a significant association between prenatal depression and differential DNAm in cg23262030 for the BDI-II continuous variable (Δ beta per BDI-II total IQR = 1.47E-03, *p*-value = 1.18E-07) (Fig. 1). This CpG site also had suggestive *p*-values for the BDI-II 14 threshold (Δ beta = 4.10E-04, *p*-value = 3.76E-06) and nominally significant p-values for the BDI-II 20 threshold, EPDS continuous, and EPDS 13 threshold variables (Table 2, Fig. 1, Figures S5–S8). Associations with DNAm in cg23262030 were similar for data from both arrays, however, the association for the 450K array was not significant (Fig. 2). For CpG sites that reached p-values less than 5 × 10^−4^ for at least one of the depression scales in the meta-analysis, beta estimates for the BDI-II and EPDS continuous variables were correlated (Figure S9).

**Table 2:** Effect sizes and *p*-values of significant CpG sites for each depression variable.

A. Primary outcomes: Maternal depression scores and newborn DNAm					
CpG Site	Chromosome: Position	Variable	Δ beta per IQR^d^	Standard Error	*P*-value
cg16473797^a^	chr20:61755045	EPDS Continuous	−1.06E-02	2.12E-04	1.04E-03
		BDI-II Continuous	−5.64E-03	9.19E-05	5.72E-05
cg23262030^a^	chr17:45918702	EPDS Continuous	1.71E-03	4.21E-05	7.87E-03
		BDI-II Continuous	1.47E-03	1.82E-05	**1.18E-07**
cg04859497^b^	chr2: 79923818	EPDS Continuous	−3.88E-02	1.05E-03	0.459
		BDI-II Continuous	−2.85E-02	4.01E-04	3.61E-07
**B. Secondary outcomes: Screening for maternal depression and newborn DNAm**					
**CpG Site**	**Chromosome: Position**	**Variable**	**Δ beta**^**e**^	**Standard Error**	***P*-value**
cg16473797^a^	chr20:61755045	EPDS Threshold 10	−1.32E-03	1.55E-03	0.39
		EPDS Threshold 13	−7.09E-03	2.38E-03	2.38E-03
		BDI-II Threshold 14	−1.10E-02	2.04E-03	**6.87E-08**
		BDI-II Threshold 20	−8.61E-03	2.29E-03	1.72E-04
cg23262030^a^	chr17:45918702	EPDS Threshold 10	6.94E-04	4.18E-04	9.7E-02
		EPDS Threshold 13	1.06E-03	4.44E-04	1.7E-02
		BDI-II Threshold 14	1.90E-03	4.10E-04	3.76E-06
		BDI-II Threshold 20	1.40E-03	5.01E-04	5.01E-03
cg04859497^b^	chr2: 79923818	EPDS Threshold 10	−9.15E-03	1.00E-02	0.359
		EPDS Threshold 13	−8.34E-03	1.08E-02	0.437
		BDI-II Threshold 14	−3.25E-02	9.15E-03	3.84E-04
		BDI-II Threshold 20	−6.42E-02	1.05E-02	**1.06E-09**^c^

^a^Results from the meta-analysis

^b^Results from the EPIC array EWAS

^c^Bonferroni threshold for meta-analysis: 1.29 × 10^−7^, for EPIC EWAS: 6.40 × 10^−8^

^d^Δ beta per IQR: This coefficient represents the increase of mean DNAm beta values per increase of one interquartile range (IQR) in the depression scores (EPDS or BDI Continuous). (IQR EPDS total for total participants = 6, IQR BDI-II total for total participants = 15.25, IQR EPDS total for EPIC array participants = 5, IQR BDI-II total for EPIC array participants = 14). Negative coefficients refer to smaller mean DNAm beta values in children of mothers with higher depression scores and positive coefficients refer to larger mean DNAm beta values in children of mothers with higher depression scores.

^e^Δ beta: This coefficient represents the mean difference of DNAm beta values between children of mothers who were screened positive for depression verses of those who were not. Negative coefficients refer to smaller mean DNAm beta values in children of mothers who were screened positive and positive coefficients refer to larger mean DNAm beta values in children of mothers who were screened positive for depression.

The DMRcate meta-analysis resulted in seven DMRs for the EPDS continuous score and seven DMRs for the BDI-II continuous score (Tables S2, S3). The DMR, chr18: 67069959–67070461, was significant for both depression variables (EPDS: max Δ beta estimate = −2.60E-03, *p*-value = 8.29E-10, BDI-II: max Δ beta estimate = −1.09E-03, *p*-value = 6.84E-05) (Table 3). The meta-analysis using dmrff resulted in one DMR for the EPDS continuous score, however, this DMR was not found with any other depression variable (Table S4).

**Table 3:** Max effect sizes and p-values from a meta-analysis using DMRcate for DMRs significant in two or more depression variables.

A. Primary outcomes: Maternal depression scores and newborn DNAm				
DMRs	# CpGs	Variable	Max Effect	*P*-value^a^
chr18: 67069959-67070461	6	EPDS Continuous	−2.60E-03	8.29E-10
		BDI-II Continuous	−1.09E-03	6.84E-05
chr7: 155174991-155175340	4	BDI-II Continuous	1.68E-03	4.36E-04
chr8: 70378380-70378994	7	BDI-II Continuous	1.22E-03	5.36E-03
chr15: 98195808-98196247	4	EPDS Continuous	−3.25E-03	1.19E-04
chr19: 18698825-18699423	9	EPDS Continuous	−3.50E-03	1.76E-04
**B. Secondary outcomes: Screening for maternal depression and newborn DNAm**				
**DMRs**	**# CpGs**	**Variable**	**Max Effect**	***P*-value**
chr18: 67069959-67070461	6	EPDS Threshold 10	−2.54E-02	2.59E-03
		EPDS Threshold 13	−5.40E-03	1.59E-02
		BDI-II Threshold 20	−2.35E-02	1.78E-06
chr7: 155174991-155175340	4	BDI-II Threshold 14	6.16E-02	1.89E-04
		BDI-II Threshold 20	3.81E-02	1.57E-02
chr8: 70378380-70378994	7	BDI-II Threshold 20	3.39E-02	3.28E-04
chr15: 98195808-98196247	4	EPDS Threshold 10	−3.47E-02	1.19E-04
chr19: 18698825-18699423	9	EPDS Threshold 10	−4.23E-02	1.01E-04

^a^Minimum Bonferroni adjusted *p*-value from CpGs forming the significant DMR.

### Secondary outcomes: Screening for maternal depression and newborn DNAm

The analyses of our secondary outcomes identified additional significant CpG sites that were also at least suggestive for our primary outcomes (Figure S10–S17). Two CpG sites, cg03489382 and cg19350511, were significant for the 450K array for the BDI-II 14 threshold variable but not for the EPIC array (Figure S10, S11). The EWAS of the EPIC data resulted in cg04859497 being statistically significant for the BDI-II threshold 20 (Δ beta = −6.42E-02, *p*-value = 1.06E-09) (Table 2, Figure S12). This CpG site is unique to the EPIC array and not available on the 450K array. The *p*-values for the CpG site were suggestive for the primary outcome BDI-II but not for the primary outcome EPDS (Table 2). The meta-analysis for both the arrays resulted in a significant association between differential DNAm in cg16473797 (BDI-II 14 threshold: Δ beta = −1.10E-02, *p*-value = 6.87E-08) and prenatal depression (Figure S6). This CpG site had suggestive p-values for the continuous BDI-II variable (Δ beta per BDI-II total IQR = −5.64E-03, *p*-value = 5.72E-05) and the BDI-II 20 threshold (Δ beta = −8.61E-03, *p*-value = 1.72E-04) and nominally significant p-values for the EPDS continuous and EPDS 13 threshold variables (Table 2). Associations with DNAm in cg16473797 were similar for data from both arrays (Fig. 2).

The DMRcate meta-analysis resulted in 24 DMRs for the binary variables (EPDS threshold-10: four DMRs, EPDS threshold-13: four DMRs, BDI-II threshold-14: eight DMRS, BDI-II threshold-20: eight DMRs) (Tables S5–S8). Five DMRs were significant for more than one variable, continuous or dichotomized, and two of the five DMRs, chr18: 67069959–67070461, chr7: 155174726–155175340, were significant for more than one dichotomized variable (Table 3). The meta-analysis using dmrff did not produce significant DMRs for the dichotomized depression variables.

## Discussion

In this study of infants from a peri-urban region in a low-resourced community in South Africa, we found prenatal depression to be associated with differential methylation in three CpG sites and within multiple DMRs measured in cord blood of newborns from the Drakenstein Child Health Study.

### Comparison with previous studies

The association between maternal prenatal depression and differences in infant DNAm is not completely understood. Previous studies measuring DNAm from cord blood have shown mixed results. A study by Viuff et al. (2018), which used the EPDS threshold-12 variable to screen for prenatal depression, found differential methylation in two CpG sites to be associated with prenatal depression while a study by Cardenas, A. et al. (2019), which used the Brief Symptom Inventory threshold-0.80 variable to screen for prenatal depression, found differential methylation in three different CpGs to be associated with prenatal depression [10, 11]. However, the significant sites for both of these studies were unable to be replicated using the Generation R study [10, 11]. The five CpG sites identified in previous studies were not significantly associated with prenatal depression in our cohort, which is in line with the results from the Generation R study (Tables S9, S10). As for DMRs, Cardenas et al. (2019) did not find any DMRs significantly associated with prenatal depression [11]. However, Viuff, et al. (2018) found 39 DMRs to be associated with prenatal depression [10]. Of these 39 DMRs, a DMR containing eight CpGs, chr8:70378380–70378995 [10], overlapped with a seven CpG DMR, chr8: 70378380–70378994 found to be significantly associated with the BDI-II continuous and BDI-II threshold-20 in our study. This replicated DMR in chr8 was previously found to be significant for mid-pregnancy maternal depression, which is defined as the depression between 18 to 32 weeks gestation [10]. This overlaps with the time maternal depression was assessed in our study, which was between 28 and 32 weeks gestation [32]. The DMR in chr8: 70378380–70378994 overlaps with the promoter region for *SULF1* which codes for the extracellular sulfatase Sulf-1 and is involved in regulating heparin sulfate (HS)-dependent signalling pathways [31]. In mice, deficiencies in *SULF1* were associated with impaired neurite outgrowth, providing evidence for the role of *SULF1* in nervous system development [33, 34]. One site within this DMR, cg07051728, was found to have a significant correlation between DNAm in brain tissue and DNAm in the blood (Table S11).

### Primary outcomes: Maternal depression scores and newborn DNAm

In our meta-analysis of overlapping CpG sites from the 450K and EPIC arrays, one CpG site, cg23262030, was significant for the BDI-II continuous depression variable (Figure S18). This CpG site is located within a promoter region near the *SCRN2*. The full role of the *SCRN2* gene does not appear to be well understood so it is unknown whether this gene or the overlapping promoter region have an important role in child development.

The meta-analysis resulted in seven DMRs for the continuous EPDS score and seven DMRs for the continuous BDI-II score with a DMR in chr18: 67069959–67070461 being significantly associated with all depression variables except for the BDI-II threshold-14 variable. This DMR overlaps with the promoter region for docking protein 6 (*DOK6)*, specifically the protein-coding transcript DOK6–001, previously shown to perform a role in Ret-mediated neurite growth [35] and nervous system development through NT-3 mediation in mice [36]. Up to now, there has not been human research for this protein and neurodevelopment. However, in human tissues, *DOK6* has been shown to have high expression in the fetal brain [35]. This DMR contained one site with a significant correlation for DNAm between brain tissue and blood (Table S10). This DMR may be important for studying adverse developmental outcomes in children born to mothers who experienced prenatal depression. The DMR, chr15: 98195808–98196247, was significantly associated with a continuous and threshold variable in the meta-analysis however no clear link between this location and adverse birth or developmental outcomes or prenatal depression has been reported in the literature.

### Secondary outcomes: Screening for maternal depression and newborn DNAm

Our secondary outcomes include dichotomized versions of our primary outcomes using different thresholds commonly used for screening for depression. For the secondary outcomes, we found two additional significant CpG sites that were also at least suggestive for our primary outcome BDI-II. The CpG site, cg16473797, was found to be significant in the meta-analysis for the BDI-II 14 threshold (Figure S19). This site does not appear to be located within a known gene or regulatory region but does share the same position as SNP rs1358065399. The major allele for this SNP is C and the minor allele is T with a minor allele frequency less than 0.01 among the global population.

A single CpG site found from the EPIC array specific analysis was significantly associated with the BDI-II threshold-20 variable and also suggestive for the other BDI-II variables (Figure S20). This site also occupies the same position as SNP, rs140401989, which has C as the major allele and T as the minor allele (minor allele frequency = 0.01–0.04 among the global population). This site is located in the second intron within *CTNNA2* which codes for the catenin alpha-2 protein which plays an important role in neurodevelopment by acting as a regulator for actin branching, with mutations in this gene associated with a neuronal migration disorder [37]. *CTNNA2* has been shown to have higher expression in the brain than most other tissues [37]. However, the CpG site, cg04859497, was not found to have a significant correlation for DNAm across brain tissue and blood (Table S11). As a result, it is unknown whether this site would also have differential methylation in cells within prenatal brain tissue due to prenatal depression.

The meta-analysis resulted in 24 DMRs for the dichotomized variables (EPDS threshold-10: four DMRs, EPDS threshold-13: four DMRs, BDI-II threshold-14: eight DMRS, BDI-II threshold-20: eight DMRs) and five of these were significant for more than one depression variable (including the replicated DMR in chr8: 70378380–70378994 discussed above). The DMR, chr7: 155174726–155175340, and chr19: 18698825–18699631 were significant for more than one depression variable, however, the connection between these regions and adverse outcomes due to prenatal depression is not clear. Overall, these sites and DMRs may be useful for investigating the biological pathway for the association between maternal prenatal depression and adverse birth and child development outcomes.

### Strengths and limitations

Our study has several strengths. Previous studies have only focused on one dichotomized depression scale [10, 11]. In our study, we used more than one scale with continuous variables as primary outcomes and dichotomized variables as secondary outcomes to reflect the complexity of depression and to validate the robustness of our findings across different depression scales. Another strength lies in the study population being of African and mixed ancestry and from a low to a middle-income country, underrepresented populations among genetic and epigenetic studies [38, 39] (Figure S21). A major contributor to the variation in DNA methylation is genetic variation. This study includes genome-wide genotype data which was used to correct for population stratification. However, genetic variation could also contribute to differential DNA methylation through methylation quantitative trait loci (meQTLs) [40]. None of the significant individual CpG sites was found to be associated meQTLs, but CpG sites within the significant DMRs were found to be associated with meQTLs (Table S12). The meQTLs could indicate a genetic contribution to the association between the DMRs and prenatal depression. Another issue that plagues EWASs is unknown confounding. This study used Cate and Bacon to control for bias and unmeasured confounding, which are state-of-the-art confounder adjustment methods based on the calculation of surrogate variables and the empirical null distribution, respectively [28].

This study does include limitations such as a relatively small study size which reduces the power to detect differences in methylation [41]. The depression scores were collected at a single time point resulting in the inability to know the complete timeframe for the onset and duration of the prenatal depression [32]. Also, this analysis is based on depression scales instead of clinical diagnosis of prenatal depression, though the depression scales are used to screen for probable depression but are not equivalent to clinical diagnosis [21]. While using more than one scale with continuous variables as primary outcomes and dichotomized variables as secondary outcomes helps to reflect the complexity of depression and to validate the robustness of our findings across different depression scales, we acknowledge that this approach also increases the chances of false-positive findings due to the burden of multiple testing. Furthermore, having two datasets measured on different array platforms resulted in EPIC specific sites not being assessed in the meta-analysis excluding these results from benefiting from larger samples sizes. Another limitation in this study is the use of heterogeneous tissue. Although the variance in the proportions of cell types was controlled for using methylation predicted estimated cell counts [42], the specific cell type origin for a given change in methylation was impossible to determine. This is an issue that is alleviated using techniques such as single-cell methylation measurements. Another limitation is the use of IMAGE-CpG to determine if there is a significant correlation between differential methylation in significant CpG sites in blood and brain tissue. The blood samples for this study were taken at birth while the samples for IMAGE-CpG have an age range of 5–61 years [31]. DNA methylation changes with age [43] which may result in incorrect associations or lack of associations for significant CpG sites in blood and brain tissue. A large limitation in most EWASs is in interpreting what changes in methylation actually mean. Even though there have been studies showing the impact of DNAm on gene expression, interpretations must be taken with caution. Additionally, many of the genes which were differentially methylated in association with maternal depression in our study have been linked to various neurological outcomes. We know that DNAm varies most greatly between tissues, as establishing cellular identity is one of the main functions of DNAm. As such, the methylation status observed in the blood cannot be extrapolated to the methylation status in the brain. While there are datasets with matched brain and blood methylation available to help support the link between blood methylation and neurological outcomes, interpretations of differential methylation need to be taken with caution.

## Conclusion

Maternal depression was associated with differential DNAm in three CpG sites and within multiple DMRs. The DMR chr8: 70378380–70378994 has been associated with maternal depression in a previous study [10]. The remaining sites and DMRs, to our knowledge, have not been previously associated with maternal depression. Further research is needed to replicate this finding and to investigate its impact on birth outcomes and child development.

## Web resources

Ensembl, NCBI’s Gene resources, Human Protein Atlas, mQTLdb:

Accessed on 4/9/2021

- Overlapping genes for DMR chr8: 70378380-70378995
- Overlapping genes for DMR chr18: 67069959-67070461
- Overlapping genes for DMR chr15: 98195808-98196247: http://grch37.ensembl.org/Homo_sapiens/Location/View?r=15:98196029-98196118;db=core
- *CTNNA2* position of cg04859497: https://grch37.ensembl.org/Homo_sapiens/Location/View?db=core;g=ENSG00000066032;r=2:79900309-79939089;t=ENST00000496558

Accessed on 8/28/2021

- Position of cg23262030 in the genome. https://grch37.ensembl.org/Homo_sapiens/Location/View?r=17:45918302-45919105;db=core
- Position of cg16473797 in the genome. https://grch37.ensembl.org/Homo_sapiens/Location/View?r=20%3A61754945-61755145
- Function of the *SCRN2* gene: https://www.genecards.org/cgi-bin/carddisp.pl?gene=SCRN2
- Overlapping genes for DMR chr7: 55174991-155175340
- Overlapping genes for DMR chr19: 18698825-18699423
- rs1358065399 genomic position and allele frequency: https://grch37.ensembl.org/Homo_sapiens/Variation/Explore?db=core;r=20:61754945-61755145;v=rs1358065399;vdb=variation;vf=440868348
- rs140401989 genomic position and allele frequency: https://grch37.ensembl.org/Homo_sapiens/Variation/Explore?db=core;r=2:79923800-79923830;v=rs140401989;vdb=variation;vf=160926159

Accessed on 8/31/2021

- Presence or absence of meQTLs at birth for CpG sites and DMRs: http://www.mqtldb.org/cgi-bin/search.cgi

## Supplementary information

Supplementary Figures S1–S21 and supplementary Tables S1–S11

Supplementary Table S12
