# Supplementary material for: Association between maternal depression during pregnancy and newborn DNA methylation
Source: Transl Psychiatry. 2021 Nov 8;11:572. doi: 10.1038/s41398-021-01697-w (PMC8576002; doi:10.1038/s41398-021-01697-w)
Supplement: Supplementary file 1 — Supplementary Figures S1–S21 and supplementary Tables S1–S11 [file 41398_2021_1697_MOESM1_ESM.pdf]

Supplementary Figures

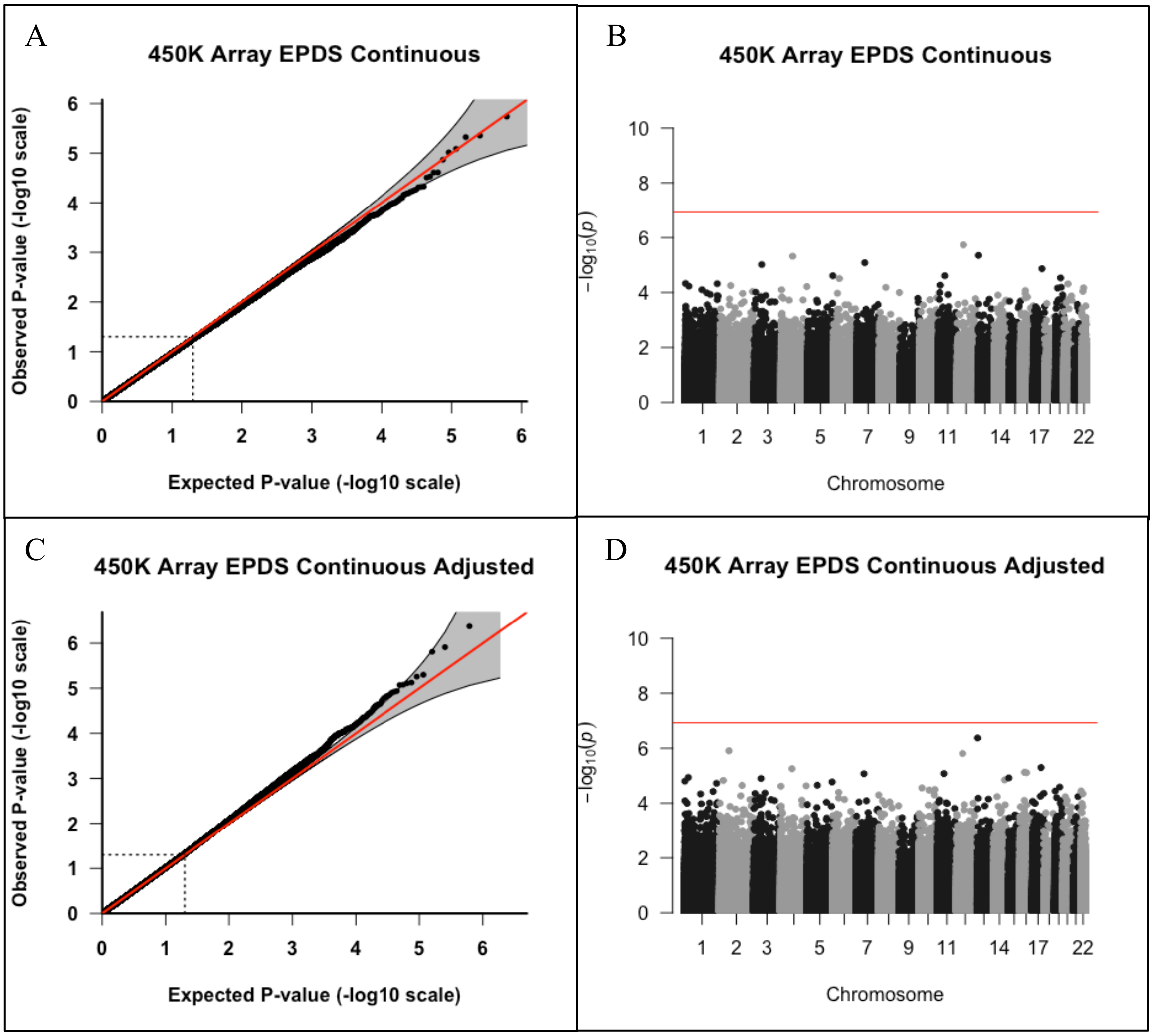

Figure S1: Results for the 450K EWAS for the EPDS continuous variable. All association models were adjusted for covariates: mother's smoking status, average household income, child's sex, gestational age at birth, first three cell type PCs, and first five genotype PCs. A) Plot A is the QQ-plot for the unadjusted p-values. B) Plot B is the Manhattan plot for the unadjusted p-values. C) Plot C is the QQ-plot for the adjusted p-values using Bacon and Cate. D) Plot D is the Manhattan plot for adjusted p-values using Bacon and Cate.

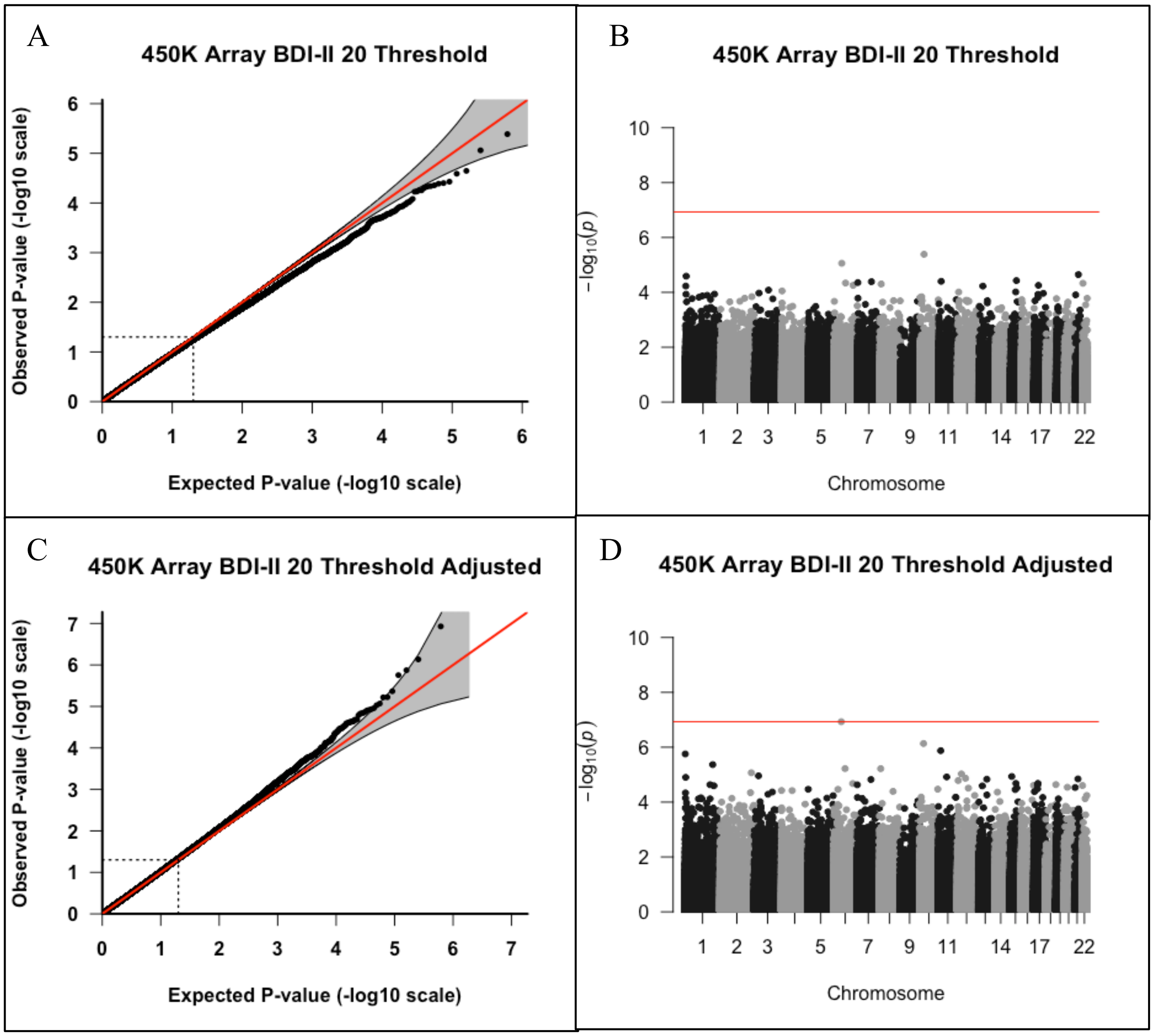

Figure S2: Results for the 450K EWAS for the BDI-II continuous variable. All association models were adjusted for covariates: mother's smoking status, average household income, child's sex, gestational age at birth, first three cell type PCs, and first five genotype PCs. A) Plot A is the QQ-plot for the unadjusted p-values. B) Plot B is the Manhattan plot for the unadjusted p-values.. C) Plot C is the QQ-plot for the adjusted p-values using Bacon and Cate. D) Plot D is the Manhattan plot for adjusted p-values using Bacon and Cate.

A

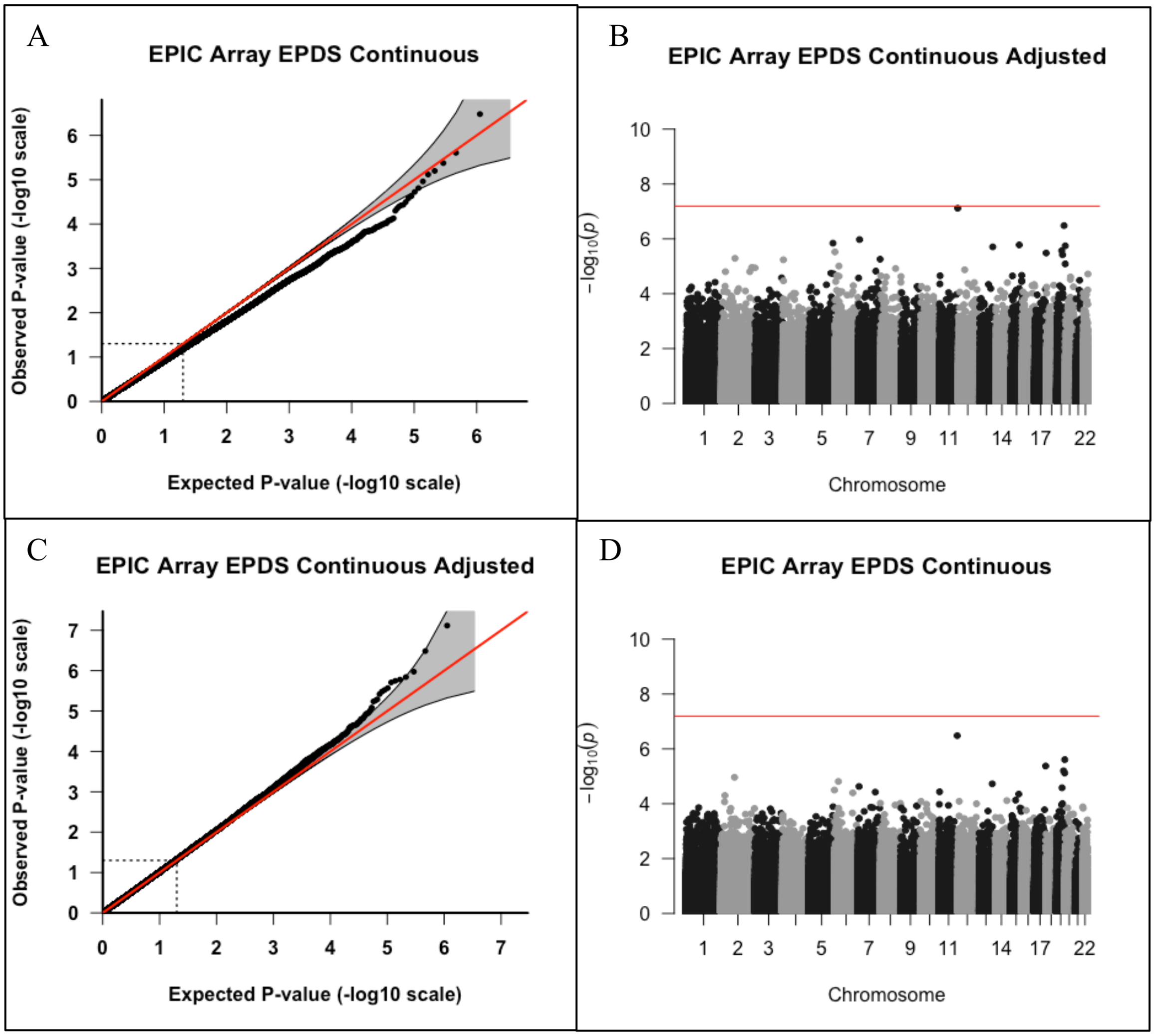

Figure S3: Results for the EPIC EWAS for the EDPS continuous variable. All association models were adjusted for covariates: mother's smoking status, average household income, child's sex, gestational age at birth, first three cell type PCs, and first five genotype PCs. A) Plot A is the QQ-plot for the unadjusted p-values. B) Plot B is the Manhattan plot for the unadjusted p-values.. C) Plot C is the QQ-plot for the adjusted p-values using Bacon and Cate. D) Plot D is the Manhattan plot for adjusted p-values using Bacon and Cate.

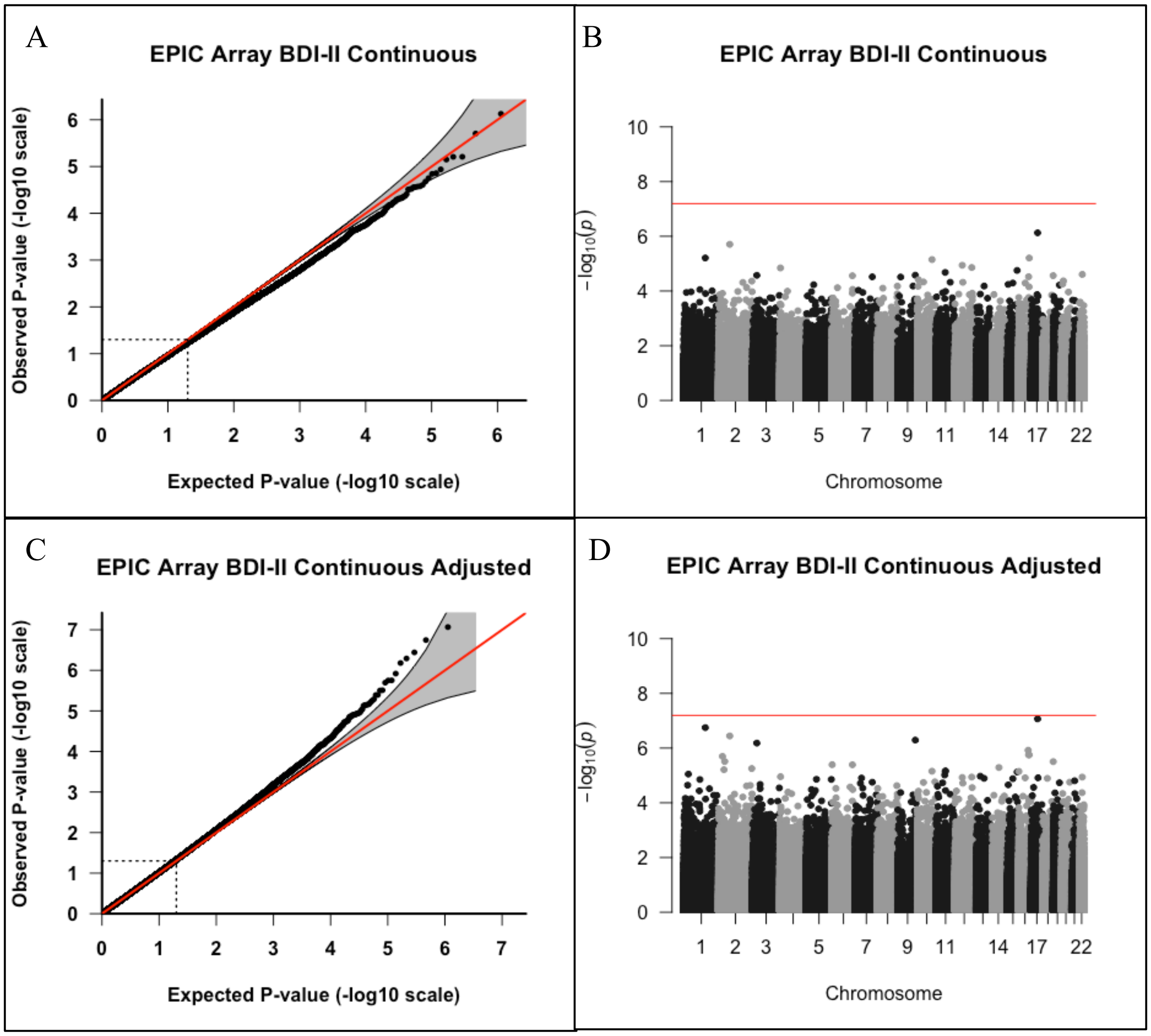

Figure S4: Results for the EPIC EWAS for the BDI-II continuous variable. All association models were adjusted for covariates: mother's smoking status, average household income, child's sex, gestational age at birth, first three cell type PCs, and first five genotype PCs. A) Plot A is the QQ-plot for the unadjusted p-values. B) Plot B is the Manhattan plot for the unadjusted p-values. The highlighted site is cg22798925. C) Plot C is the QQ-plot for the adjusted p-values using Bacon and Cate. D) Plot D is the Manhattan plot for adjusted p-values using Bacon and Cate. The highlighted site is cg22798925.

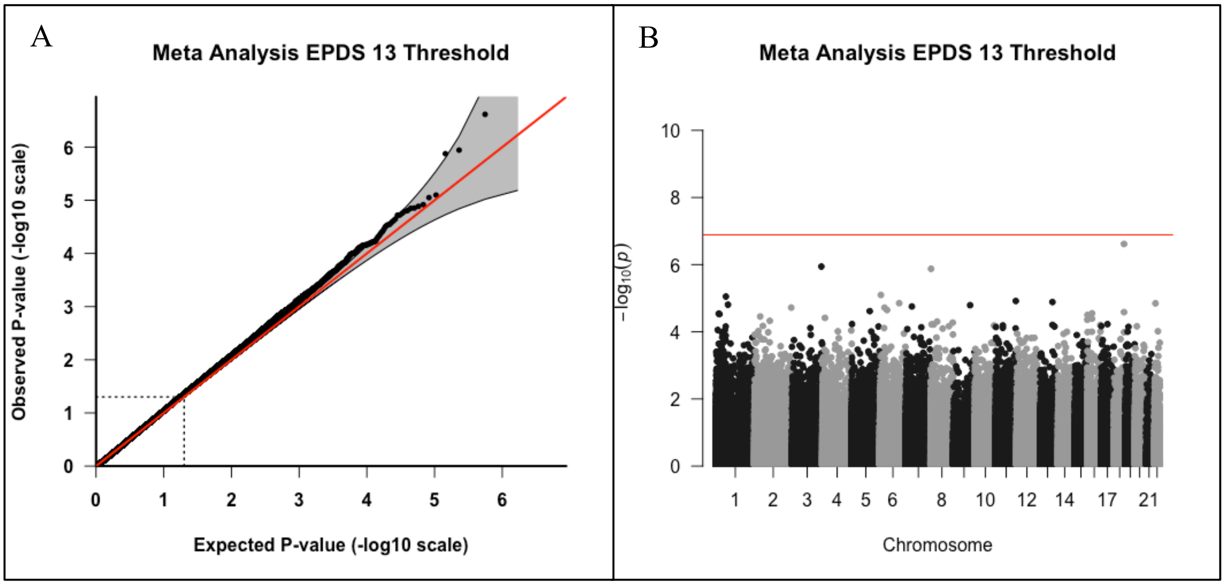

Figure S5: Results for the meta-analysis for the EPDS 13 threshold variable. The meta-analysis was adjusted for all covariates (mother's smoking status, average household income, child's sex, gestational age at birth, first three cell type PCs, and first five genotype PCs) and the p-values were adjusted for unmeasured confounding using Bacon and Cate prior to the meta-analysis. A) Plot A is the QQ-plot for the p-values. B) Plot B is the Manhattan plot for the p-values.

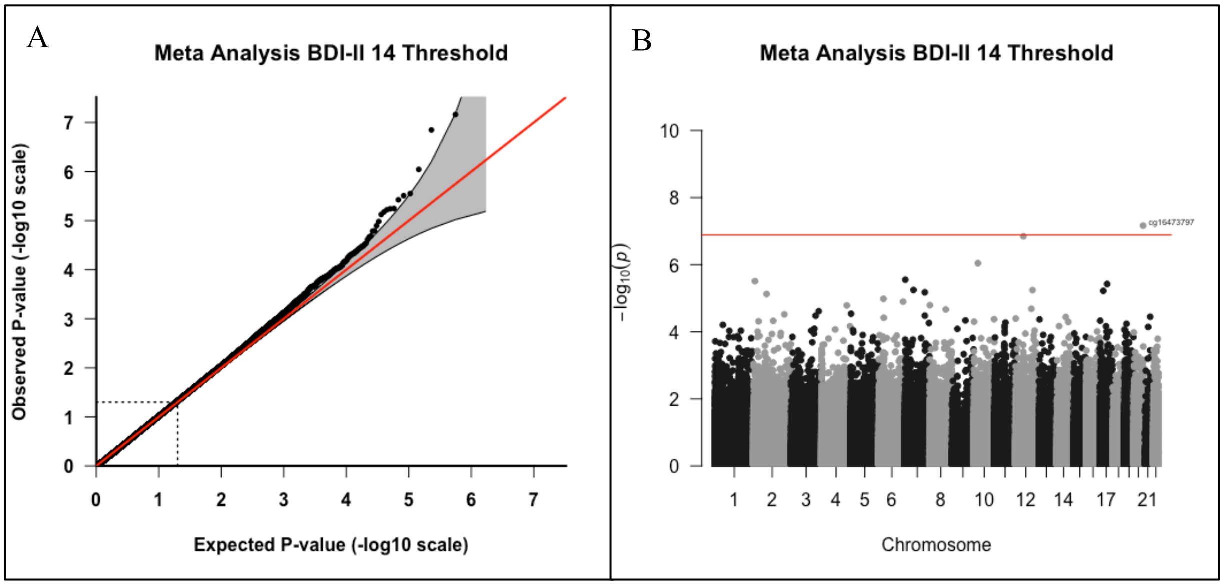

Figure S6: Results for the meta-analysis for the BDI-II 14 threshold variable. The meta-analysis was adjusted for all covariates (mother's smoking status, average household income, child's sex, gestational age at birth, first three cell type PCs, and first five genotype PCs) and the p-values were adjusted for unmeasured confounding using Bacon and Cate prior to the meta-analysis. A) Plot A is the QQ-plot for the p-values. B) Plot B is the Manhattan plot for the p-values.

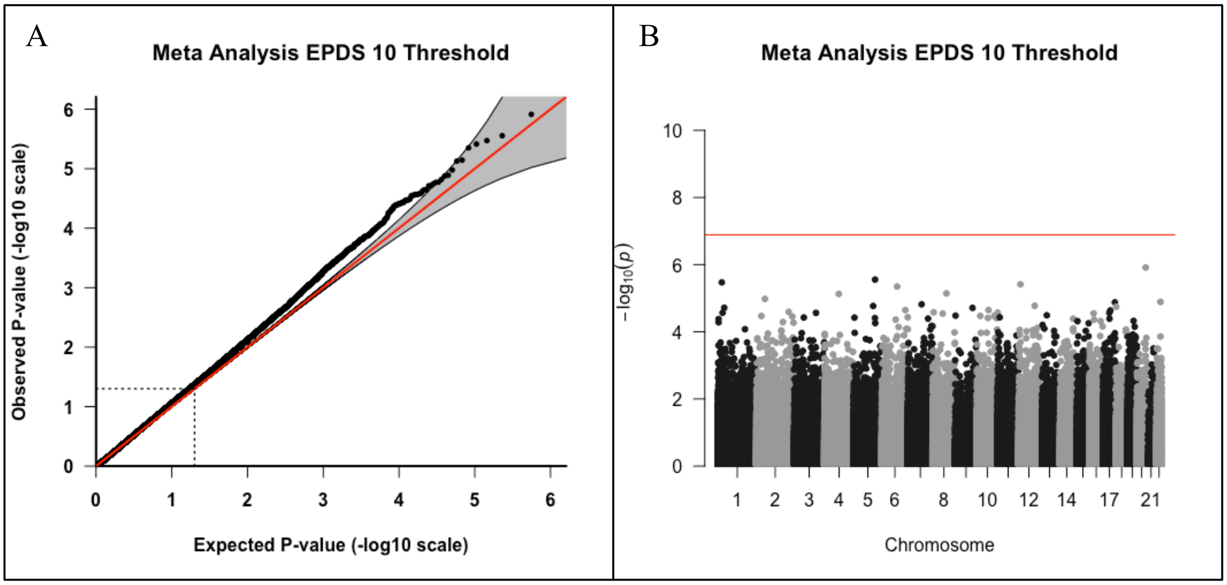

Figure S7: Results for the meta-analysis for the EPDS 10 threshold variable. The meta-analysis was adjusted for all covariates (mother's smoking status, average household income, child's sex, gestational age at birth, first three cell type PCs, and first five genotype PCs) and the p-values were adjusted for unmeasured confounding using Bacon and Cate prior to the meta-analysis. A) Plot A is the QQ-plot for the p-values. B) Plot B is the Manhattan plot for the p-values.

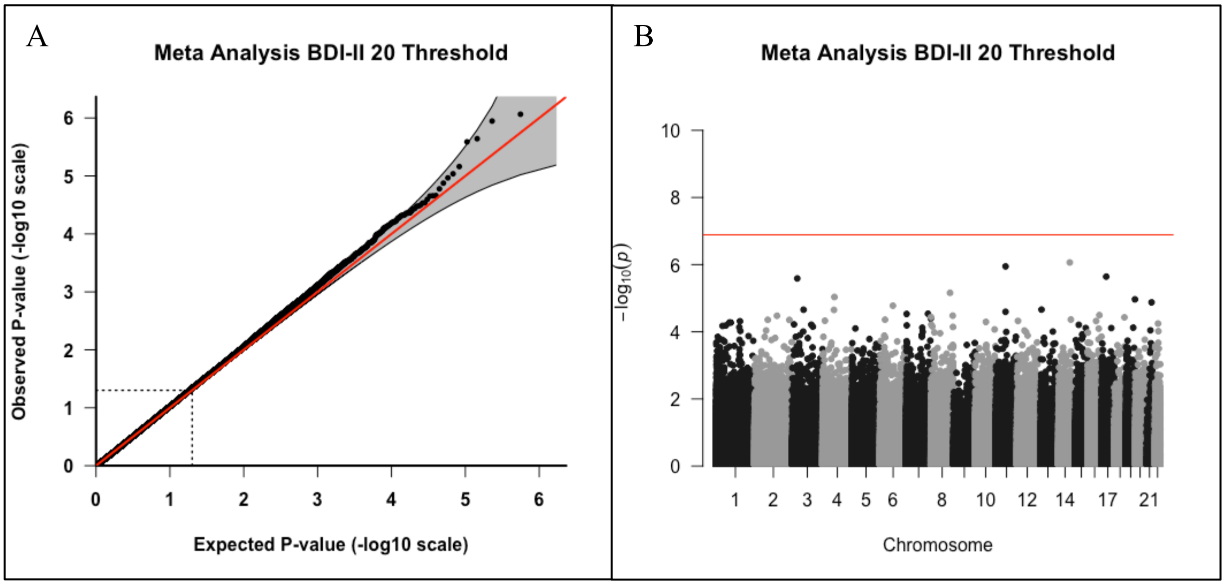

Figure S8: Results for the meta-analysis for the BDI-II 20 threshold variable. The meta-analysis was adjusted for all covariates (mother's smoking status, average household income, child's sex, gestational age at birth, first three cell type PCs, and first five genotype PCs) and the p-values were adjusted for unmeasured confounding using Bacon and Cate prior to the meta-analysis. A) Plot A is the QQ-plot for the p-values. B) Plot B is the Manhattan plot for the p-values.

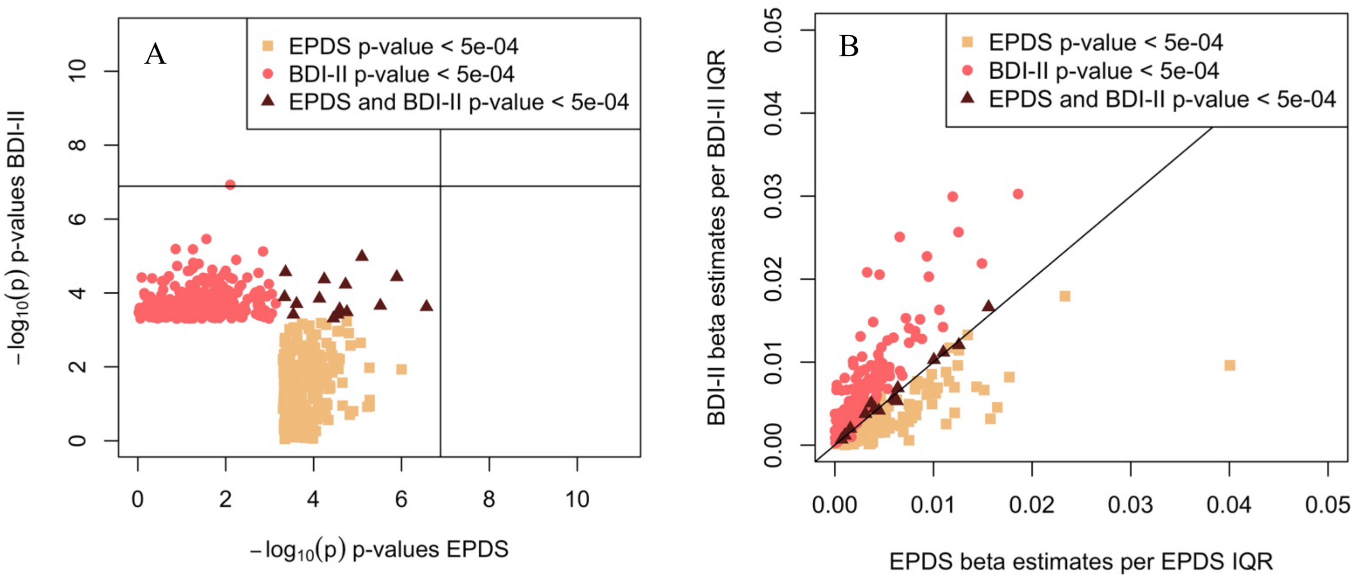

Figure S9: Comparison between the p-values and beta estimates for the EPDS and BDI-II continuous variables from the meta-analysis. The meta-analysis was adjusted for mother's smoking status, average household income, child's sex, gestational age at birth, first three cell type PCs, and first five genotype PCs. Unmeasured confounding and bias were adjusted with Cate and Bacon R packages. A) Plot A is for the p-values between the EPDS and BDI-II continuous variables below a threshold of 5e-04. B) Plot B is for the beta estimates per IQR for the EPDS and BDI-II continuous variables. The plotted values are the beta estimates multiplied by the IQR to account for the different ranges between the EPDS and BDI-II scales.

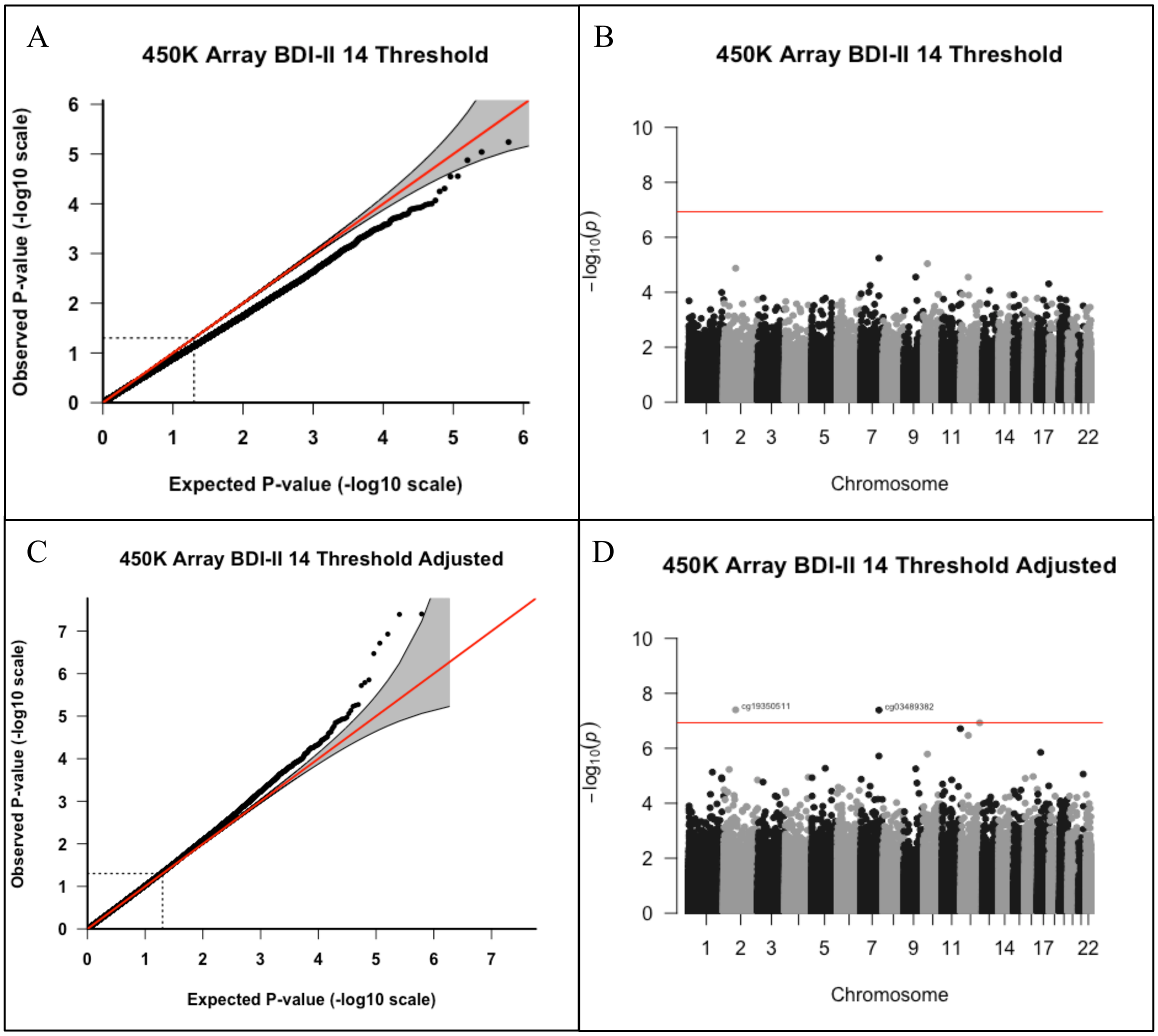

Figure S10: Results for the 450K EWAS for the BDI-II 14 threshold variable. All association models were adjusted for covariates: mother's smoking status, average household income, child's sex, gestational age at birth, first three cell type PCs, and first five genotype PCs. A) Plot A is the QQ-plot for the unadjusted p-values. B) Plot B is the Manhattan plot for the unadjusted p-values. C) Plot C is the QQ-plot for the adjusted p-values using Bacon and Cate. D) Plot D is the Manhattan plot for adjusted p-values using Bacon and Cate.

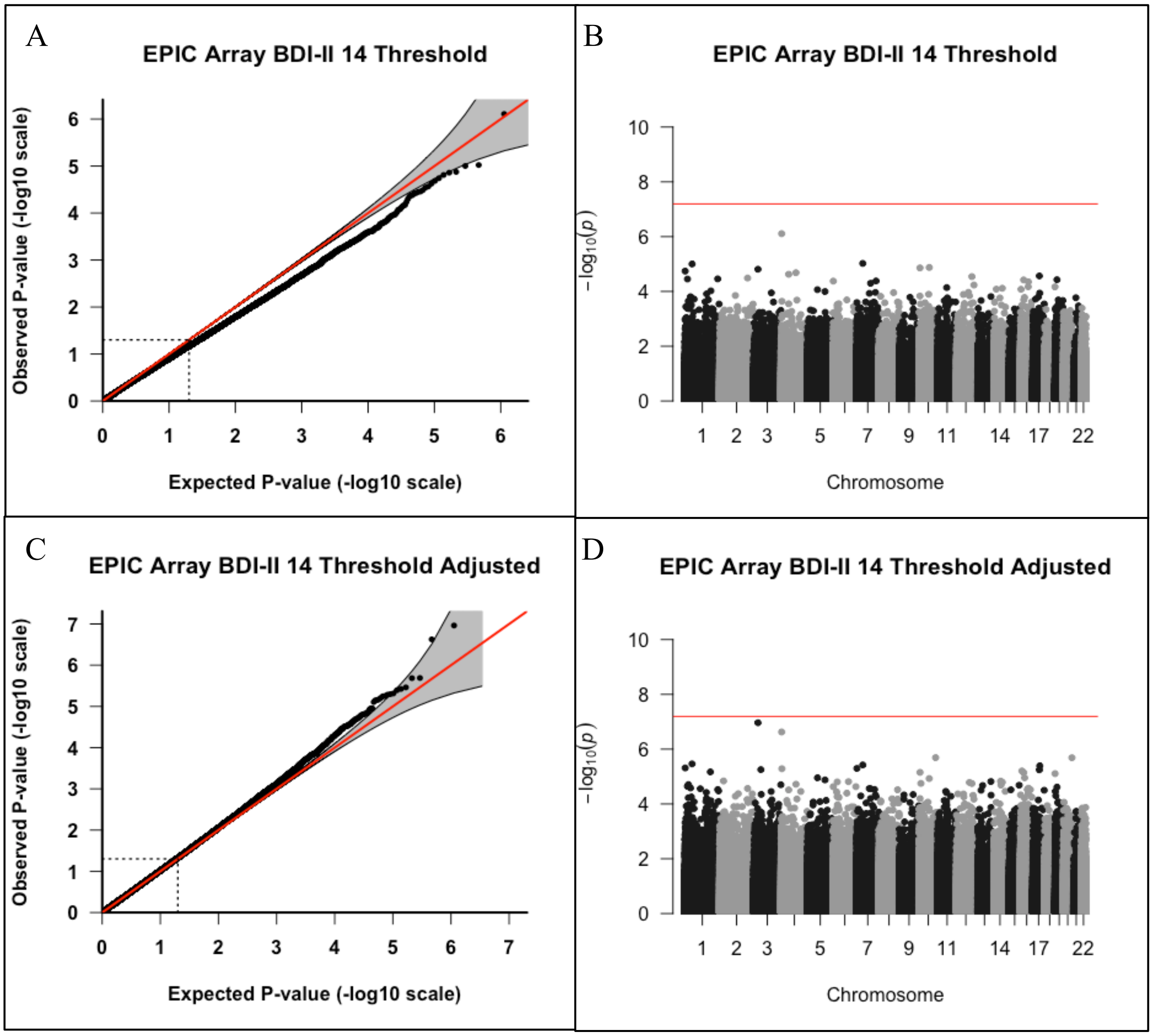

Figure S11: Results for the EPIC EWAS for the BDI-II 14 threshold variable. All association models were adjusted for covariates: mother's smoking status, average household income, child's sex, gestational age at birth, first three cell type PCs, and first five genotype PCs. A) Plot A is the QQ-plot for the unadjusted p-values. B) Plot B is the Manhattan plot for the unadjusted p-values. C) Plot C is the QQ-plot for the adjusted p-values using Bacon and Cate. D) Plot D is the Manhattan plot for adjusted p-values using Bacon and Cate.

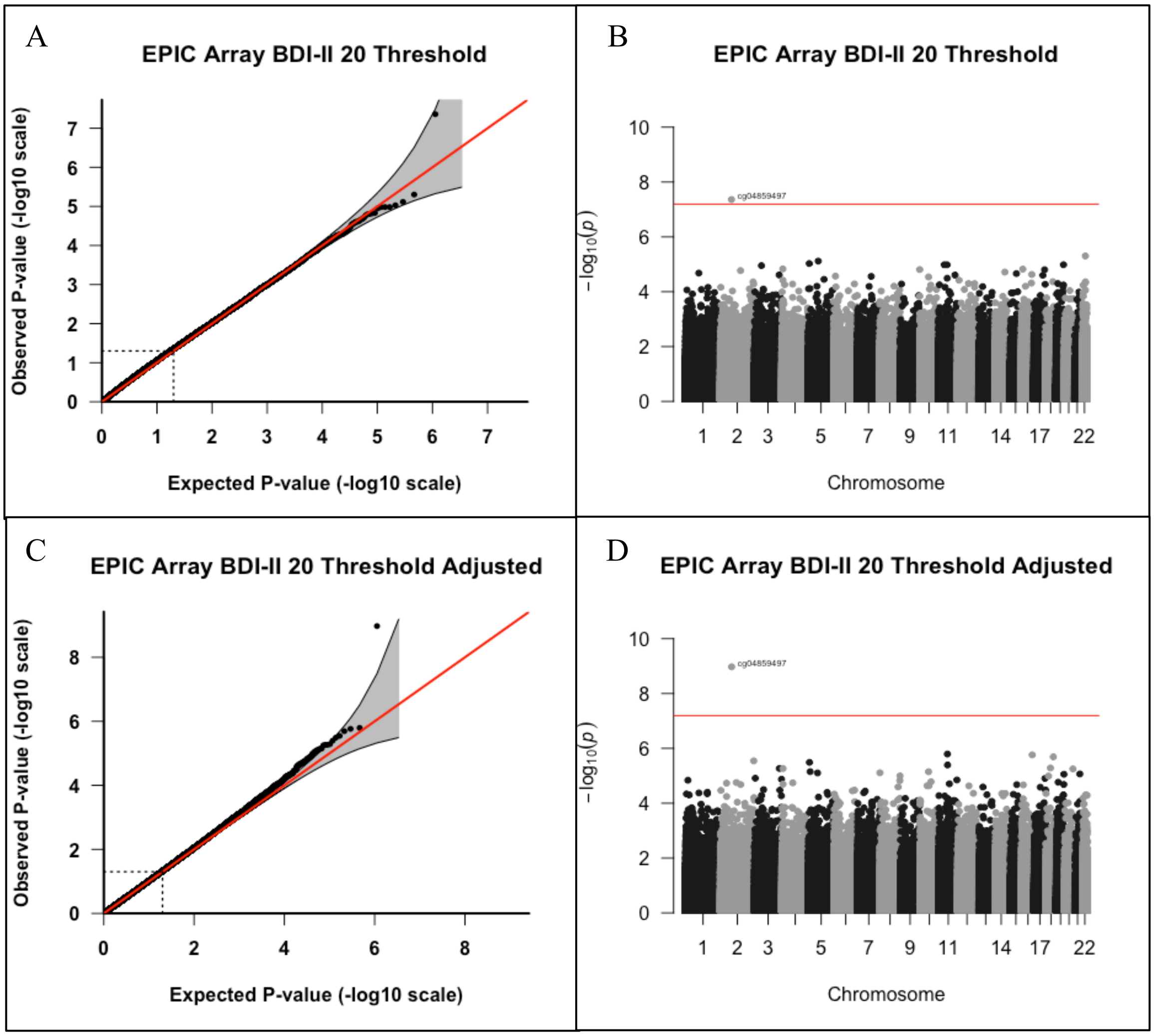

Figure S12: Results for the EPIC EWAS for the BDI-II 20 threshold variable. All association models were adjusted for covariates: mother's smoking status, average household income, child's sex, gestational age at birth, first three cell type PCs, and first five genotype PCs. A) Plot A is the QQ-plot for the unadjusted p-values. B) Plot B is the Manhattan plot for the unadjusted p-values. C) Plot C is the QQ-plot for the adjusted p-values using Bacon and Cate. D) Plot D is the Manhattan plot for adjusted p-values using Bacon and Cate.

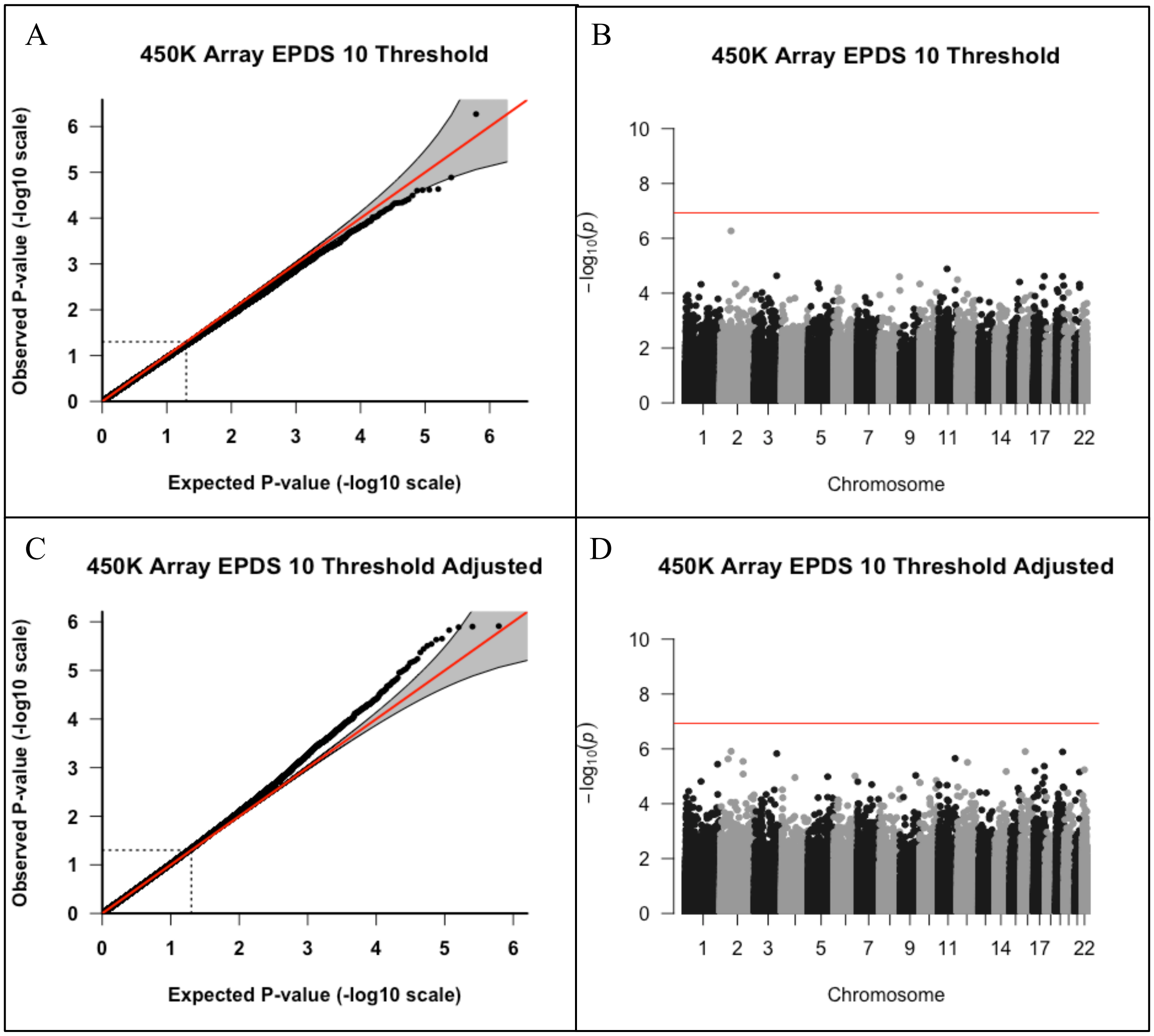

Figure S13: Results for the 450K EWAS for the EPDS 10 threshold variable. All association models were adjusted for covariates: mother's smoking status, average household income, child's sex, gestational age at birth, first three cell type PCs, and first five genotype PCs. A) Plot A is the QQ-plot for the unadjusted p-values. B) Plot B is the Manhattan plot for the unadjusted p-values. C) Plot C is the QQ-plot for the adjusted p-values using Bacon and Cate. D) Plot D is the Manhattan plot for adjusted p-values using Bacon and Cate.

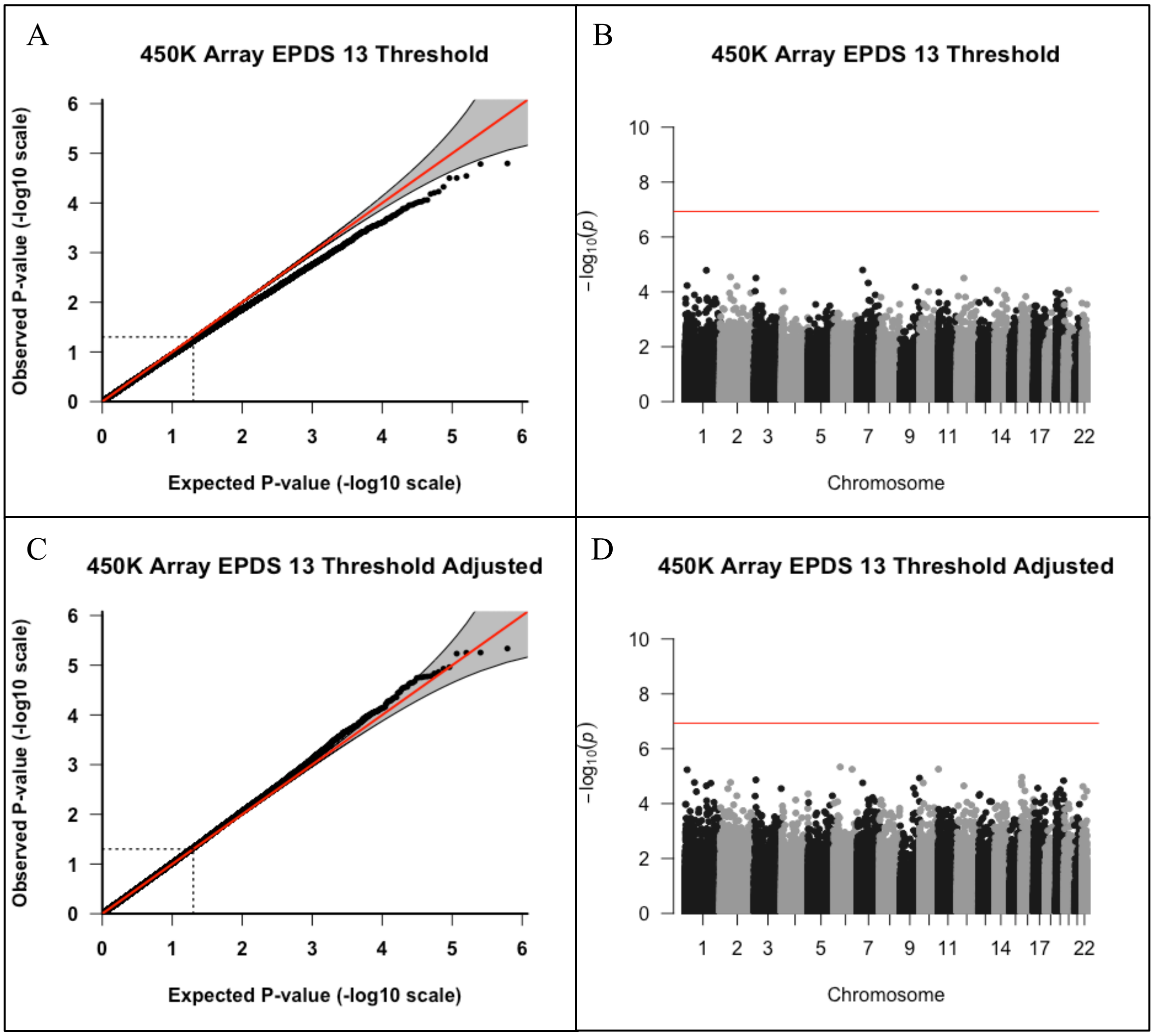

Figure S14: Results for the 450K EWAS for the EPDS 13 threshold variable. All association models were adjusted for covariates: mother's smoking status, average household income, child's sex, gestational age at birth, first three cell type PCs, and first five genotype PCs. A) Plot A is the QQ-plot for the unadjusted p-values. B) Plot B is the Manhattan plot for the unadjusted p-values. C) Plot C is the QQ-plot for the adjusted p-values using Bacon and Cate. D) Plot D is the Manhattan plot for adjusted p-values using Bacon and Cate.

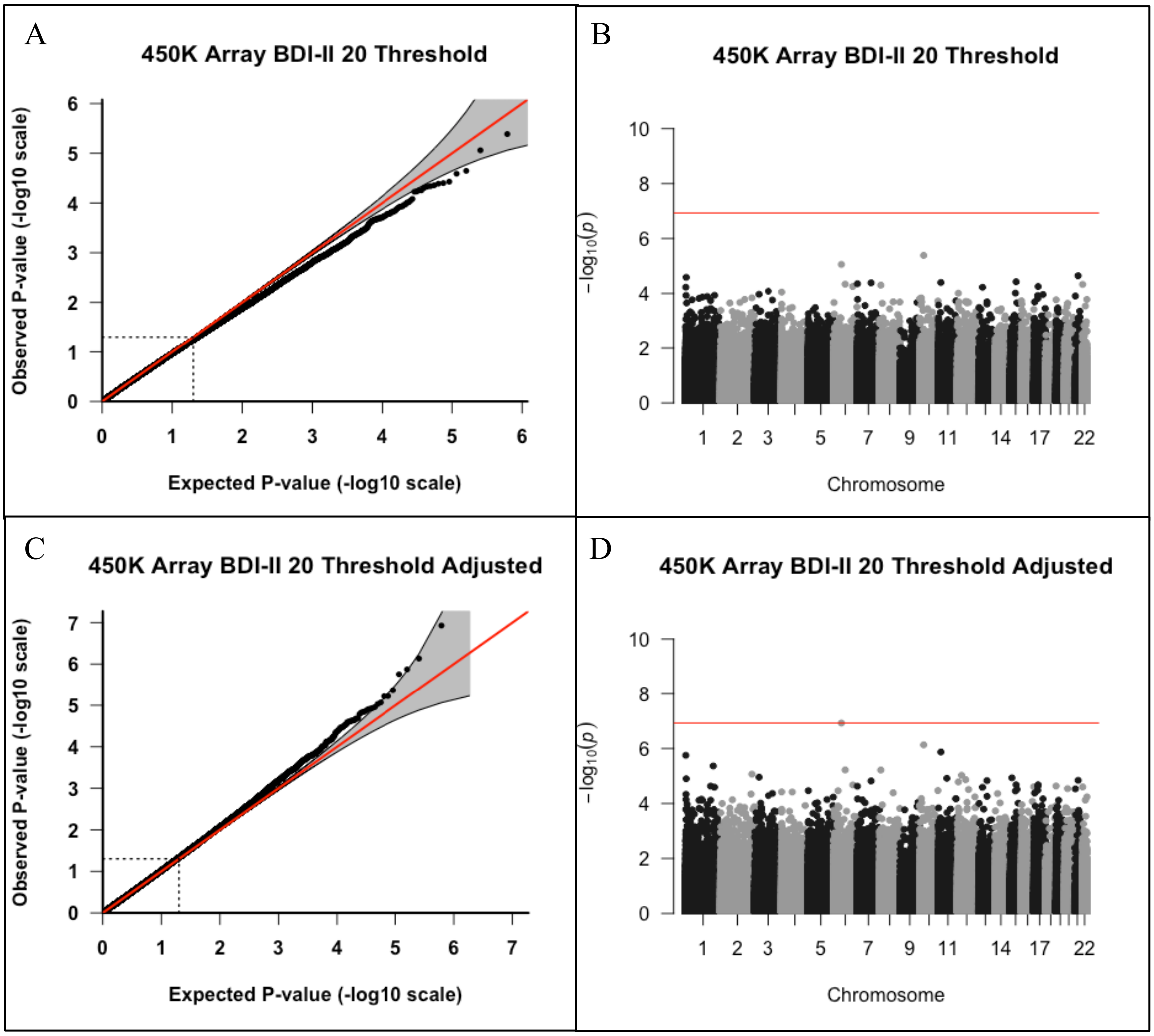

Figure S15: Results for the 450K EWAS for the BDI-II 20 threshold variable. All association models were adjusted for covariates: mother's smoking status, average household income, child's sex, gestational age at birth, first three cell type PCs, and first five genotype PCs. A) Plot A is the QQ-plot for the unadjusted p-values. B) Plot B is the Manhattan plot for the unadjusted p-values. C) Plot C is the QQ-plot for the adjusted p-values using Bacon and Cate. D) Plot D is the Manhattan plot for adjusted p-values using Bacon and Cate.

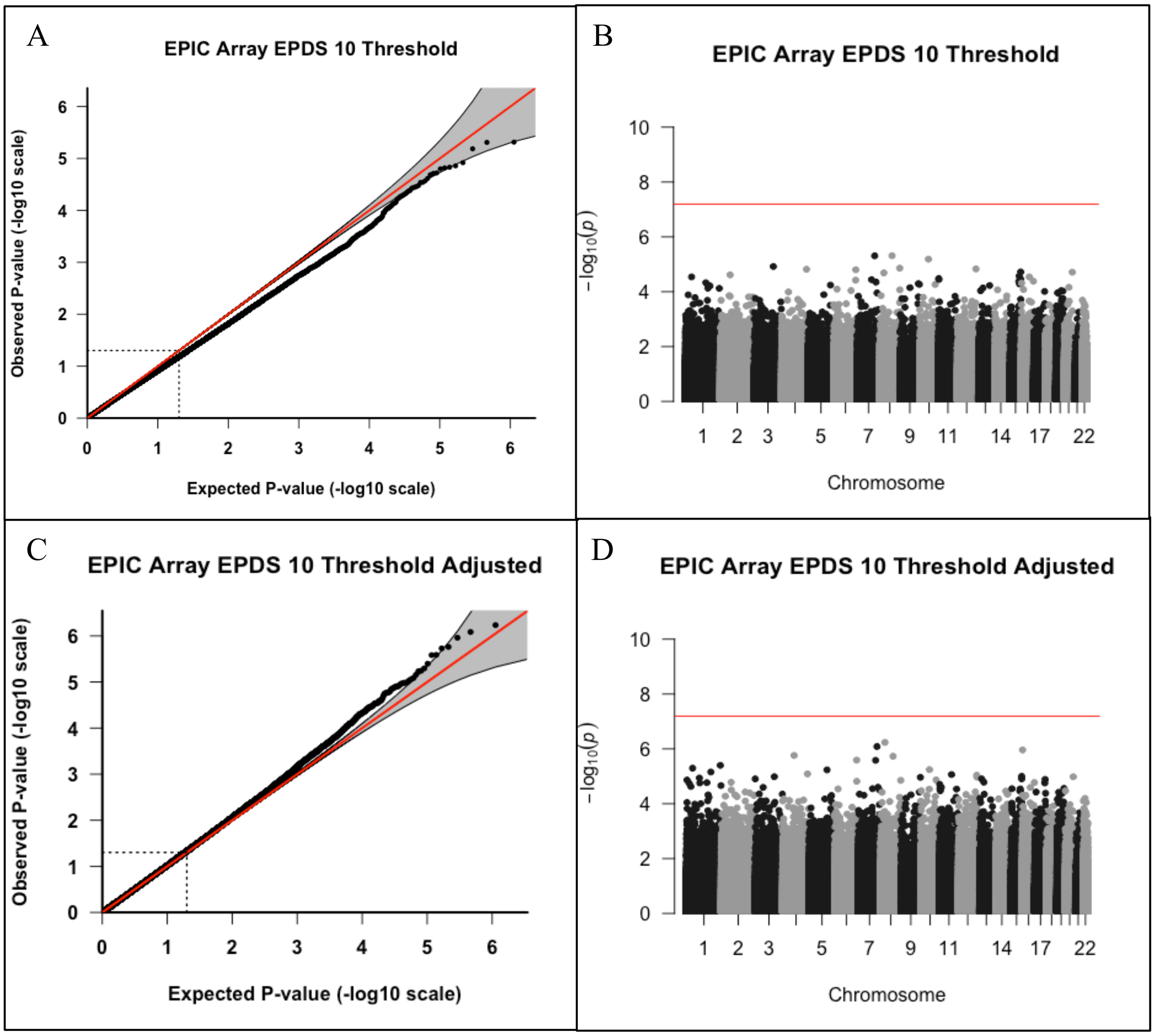

Figure S16: Results for the EPIC EWAS for the EPDS 10 threshold variable. All association models were adjusted for covariates: mother's smoking status, average household income, child's sex, gestational age at birth, first three cell type PCs, and first five genotype PCs. A) Plot A is the QQ-plot for the unadjusted p-values. B) Plot B is the Manhattan plot for the unadjusted p-values. C) Plot C is the QQ-plot for the adjusted p-values using Bacon and Cate. D) Plot D is the Manhattan plot for adjusted p-values using Bacon and Cate.

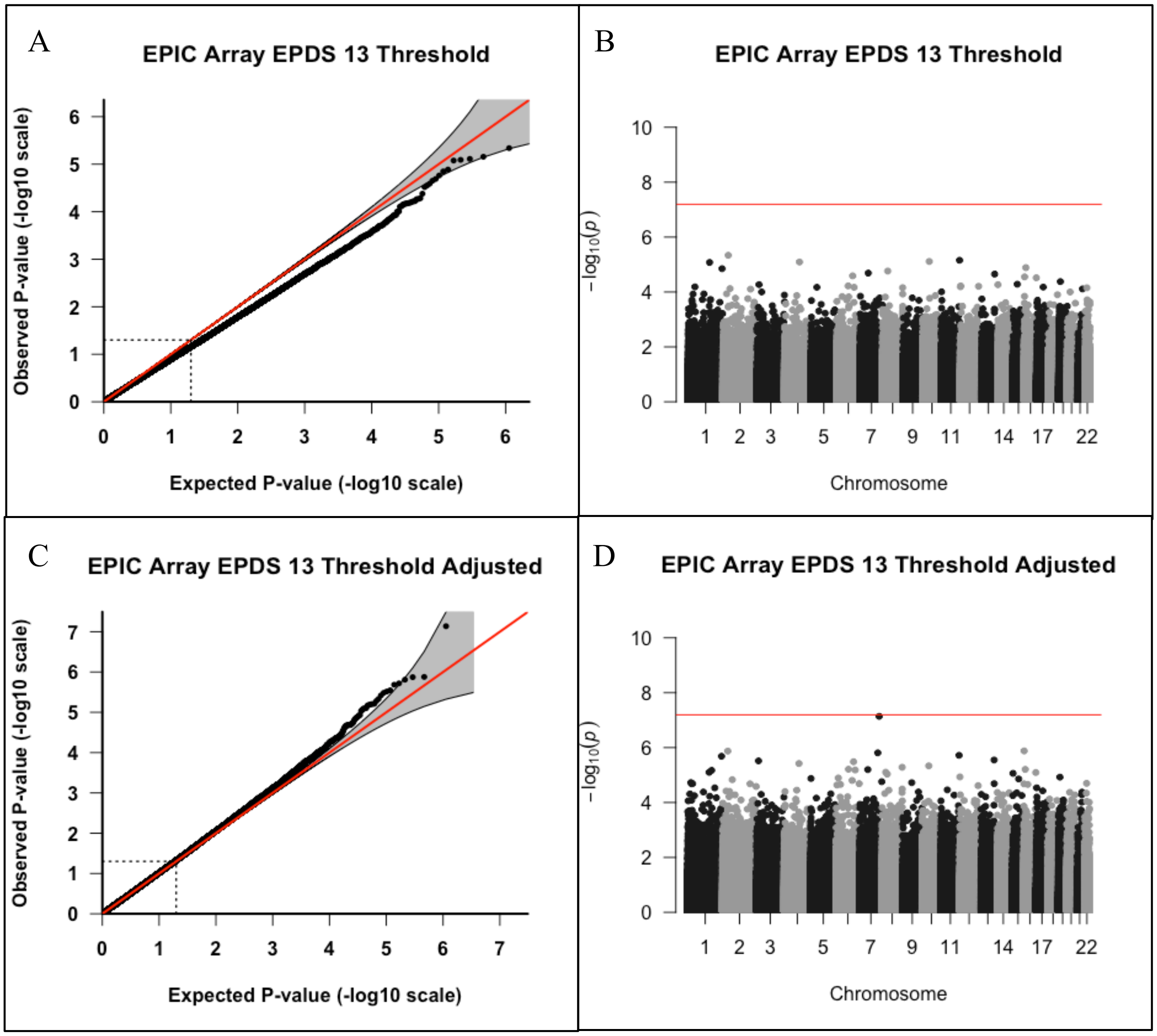

Figure S17: Results for the EPIC EWAS for the EPDS 13 threshold variable. All association models were adjusted for covariates: mother's smoking status, average household income, child's sex, gestational age at birth, first three cell type PCs, and first five genotype PCs. A) Plot A is the QQ-plot for the unadjusted p-values. B) Plot B is the Manhattan plot for the unadjusted p-values. C) Plot C is the QQ-plot for the adjusted p-values using Bacon and Cate. D) Plot D is the Manhattan plot for adjusted p-values using Bacon and Cate.

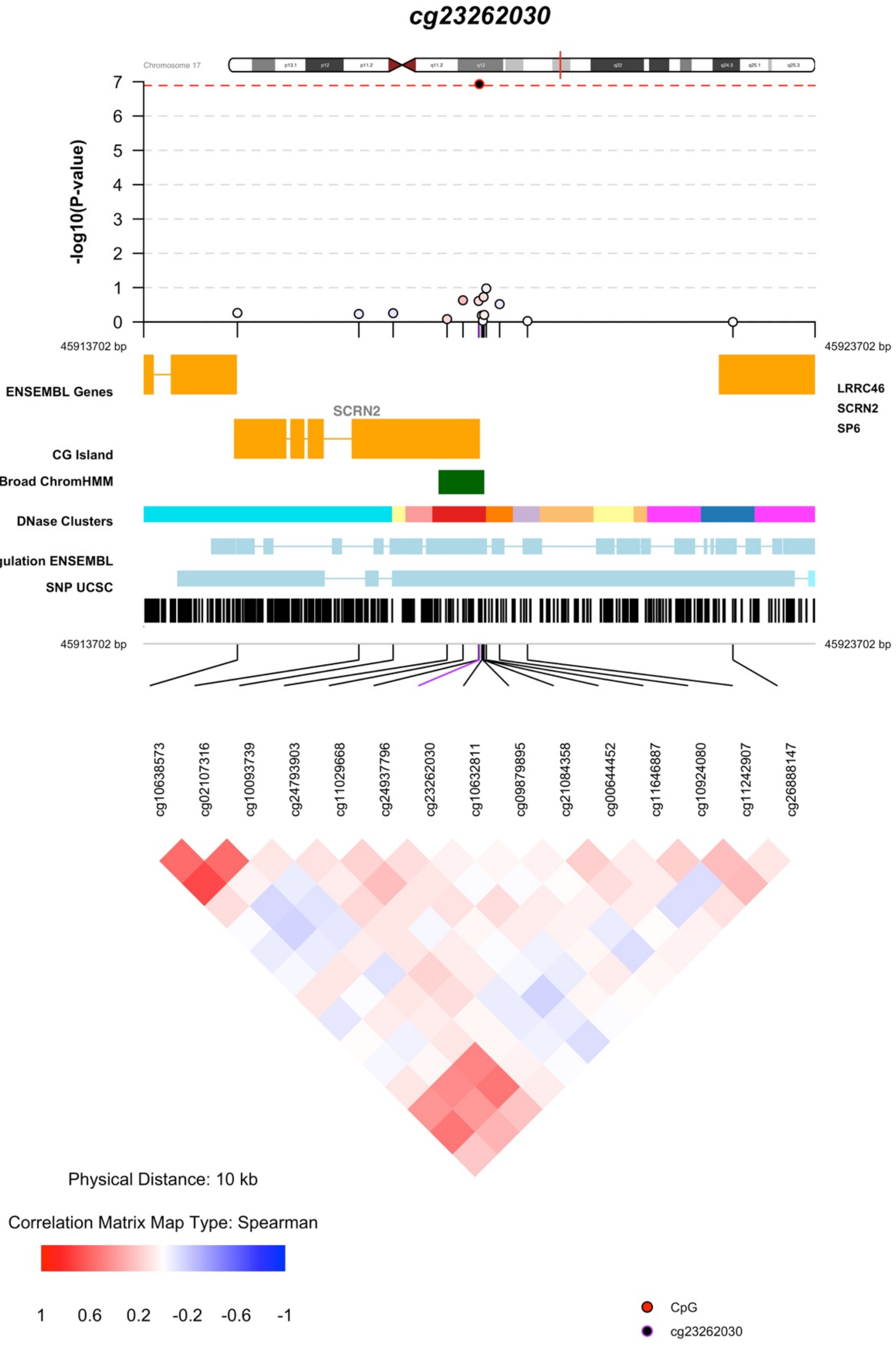

Figure S18: CoMET results for cg23262030. The CoMET results were obtained using p-values from the meta-analysis for the BDI-II 20 continuous variable while adjusting for all covariates. The CpG sites include sites 5000 bp upstream and downstream cg23262030.

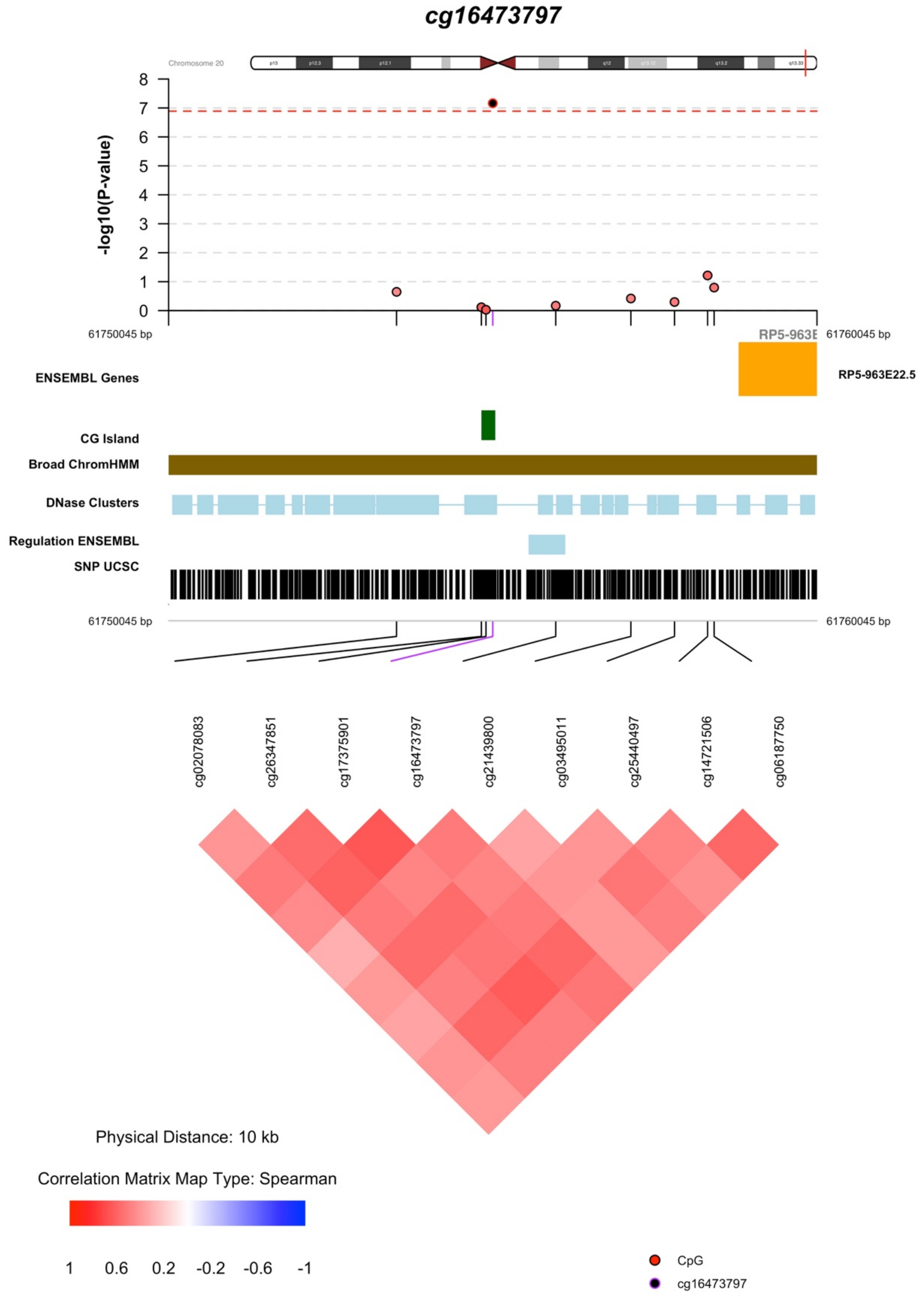

Figure S19: CoMET results for cg16473797. The CoMET results were obtained using p-values from the meta-analysis for the BDI-II 14 threshold variable while adjusting for all covariates. The CpG sites include sites 5000 bp upstream and downstream cg16473797.

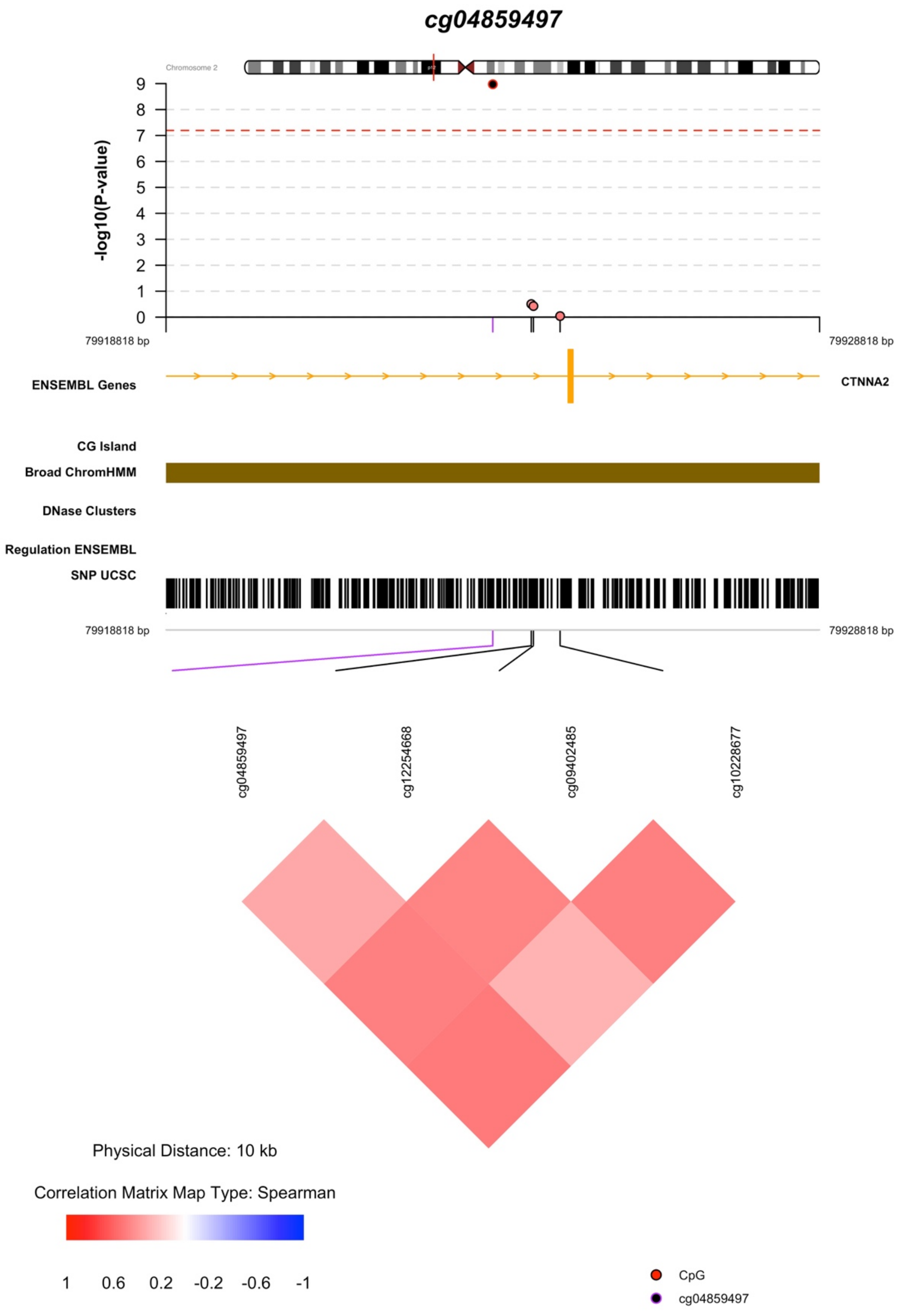

Figure S20: CoMET results for cg04859497. The CoMET results were obtained using p-values from the EPIC EWAS for the BDI-II 20 threshold variable while adjusting for all covariates. The CpG sites include sites 5000 bp upstream and downstream cg04859497.

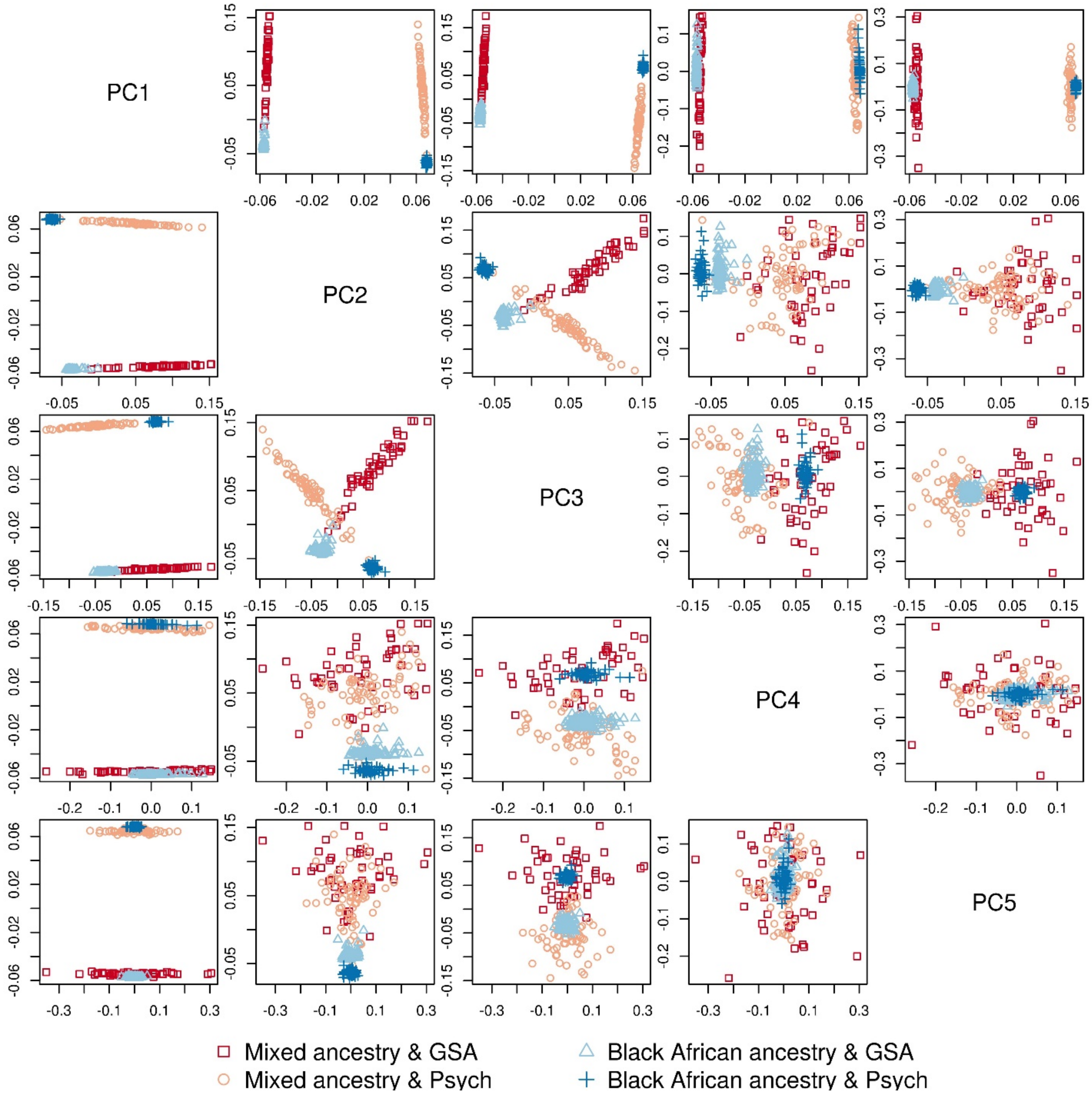

Figure S21. Correlation between the first five principle components (PC1-PC5). Ancestry and genotyping are shown in the legend.

Supplementary Tables

Table S1: Sensitivity analysis results for the significant single CpG sites with and without HIV exposure as a covariate

		Without HIV exposed[a]		With HIV exposed[a]	
CpG sites	Depression Variable	Effect Δ beta[b]	p-value	Effect Δ beta[b]	p-value
cg16473797	BDI-II 14 Threshold	-1.10E-02	6.87E-08	-1.09E-02	7.88E-08
cg23262030	BDI-II Continuous	9.62E-05	1.18E-07	9.80E-05	9.46E-08
cg04859497	BDI-II 20 Threshold	-6.42E-02	1.06E-09	-6.43E-02	1.16E-09

[a] - All association models were adjusted for covariates: mother's smoking status, average household income, child's sex, gestational age at birth, first three cell type PCs, and first five genotype PCs.

[b] - Δ beta: This coefficient represents the mean difference of DNAm beta values between children of mothers who were screened positive for depression versus of those who were not. Negative coefficients refer to smaller mean DNAm beta values in children of mothers who were screened positive and positive coefficients refer to larger mean DNAm beta values in children of mothers who were screened positive for depression.

Table S2: DMR results for DMRcate meta-analysis EPDS continuous variable

DMRs	# CpGs	P-Value[a]	Max Effect	Overlapping Promoters
**chr15:98195808-98196247**	4	1.19E-04	-3.25E-03	NA
**chr18:67069959-67070461**	6	8.29E-10	-2.60E-03	DOK6-001
**chr7:116139180-116139477**	7	2.15E-02	1.77E-03	CAV2-001, CAV2-019, CAV2-023, AC002066.1-003, CAV2-002
**chr19:18698825-18699423**	7	1.76E-04	-3.50E-03	C19orf60-001, C19orf60-002, C19orf60-007, C19orf60-005, C19orf60-008, C19orf60-004, C19orf60-003, C19orf60-006
**chr17:6899207-6899758**	9	7.30E-03	-3.25E-03	ALOX12-001, ALOX12-003, RP11-589P10.5-001
**chr14:24422419-24423864**	11	7.46E-08	-2.00E-03	DHRS4-201, DHRS4-002, DHRS4-202, DHRS4-203, DHRS4-001, DHRS4-003, DHRS4-AS1-004, DHRS4-204, DHRS4-004, DHRS4-AS1-003, DHRS4-AS1-005, DHRS4-AS1-002, DHRS4-AS1-007, DHRS4-005, DHRS4-007, DHRS4-006
**chr6:33282418-33283317**	25	3.10E-06	-1.52E-03	TAPBP-008, TAPBP-001, TAPBP-003, TAPBP-209, TAPBP-010, TAPBP-007, TAPBP-004, TAPBP-002, TAPBP-006

[a] - Adjusted with Bonferroni correction

Table S3: DMR results for DMRcate meta-analysis BDI-II continuous variable

DMRs	# CpGs	P-Value[a]	Max Effect	Overlapping Promoters
**chr2:240884831-240884925**	2	1.78E-02	1.96E-03	NA
**chr7:155174991-155175340**	3	4.36E-04	1.68E-03	AC008060.7-001
**chr5:176047151-176047485**	2	3.91E-02	9.24E-04	NA
**chr18:67069959-67070461**	6	6.84E-05	-1.09E-03	DOK6-001
**chr8:70378380-70378994**	7	5.36E-03	1.22E-03	SULF1-201, SULF1-001, SULF1-008, SULF1-009, SULF1-010
**chr10:70321574-70322442**	7	1.23E-04	1.64E-03	TET1-001
**chr6:33047944-33049360**	16	5.39E-12	2.11E-03	HLA-DPB1-002, HLA-DPA1-004, HLA-DPA1-001, HLA-DPB1-008, RPL32P1-001, HLA-DPA1-002, HLA-DPB1-006, HLA-DPA1-005, HLA-DPB1-009, HLA-DPB1-005, HLA-DPB1-007

[a] - Adjusted with Bonferroni correction

Table S4: DMR results for dmrff meta-analysis EPDS continuous variable

DMRS	# CpGs	P-Value[a]	Beta Estimate
**chr12:14996515-14996587**	5	1.43E-04	2.26E-02

[a] - Adjusted with Bonferroni correction

Table S5: DMR results for DMRcate meta-analysis BDI-II threshold-14 variable

DMRs	# CpGs	P-Value[a]	Max Effect	Overlapping Promoters
**chr7:155174991-155175340**	3	2.20E-04	3.53E-02	AC008060.7-001
**chr11:65190825-65190999**	3	1.89E-04	6.16E-02	NEAT1-002, NEAT1-001, NEAT1-202
**chr3:65342216-65342644**	3	2.19E-02	2.62E-02	NA
**chr20:32856747-32857227**	6	6.55E-05	2.77E-02	NA
**chr17:41739130-41739326**	4	9.71E-03	1.45E-02	MEOX1-001, MEOX1-201, MEOX1-003, MEOX1-002
**chr12:104697193-104697983**	12	9.90E-08	2.75E-02	EID3-001
**chr6:33047944-33048879**	15	4.59E-08	4.61E-02	HLA-DPB1-002, HLA-DPA1-004, HLA-DPA1-001, HLA-DPB1-008, RPL32P1-001, HLA-DPA1-002, HLA-DPB1-006, HLA-DPA1-005, HLA-DPB1-009, HLA-DPB1-005, HLA-DPB1-007
**chr7:27183643-27184853**	29	1.79E-03	2.54E-02	HOXA5-001, HOXA-AS3-001, HOXA5-002

[a] - Adjusted with Bonferroni correction

Table S6: DMR results for DMRcate meta-analysis EPDS threshold-10 variable

DMRs	# CpGs	P-Value[a]	Max Effect	Overlapping Promoters
**chr15:98195808-98196247**	4	1.19E-04	-3.47E-02	NA
**chr18:67069959-67070461**	6	2.79E-03	-2.54E-02	DOK6-001
**chr14:55907374-55907501**	6	3.37E-02	2.24E-02	TBPL2-001
**chr19:18698825-18699423**	7	1.01E-04	-4.23E-02	C19orf60-001, C19orf60-002, C19orf60-007, C19orf60-005, C19orf60-008, C19orf60-004, C19orf60-003, C19orf60-006

[a] - Adjusted with Bonferroni correction

Table S7: DMR results for DMRcate meta-analysis EPDS threshold-13 variable

DMRs	# CpGs	P-Value[a]	Max Effect	Overlapping Promoters
**chr18:67069959-67070461**	6	1.84E-10	-2.34E-02	DOK6-001
**chr14:24423199-24423483**	2	1.59E-02	-5.40E-03	DHRS4-201, DHRS4-002, DHRS4-202, DHRS4-203, DHRS4-001, DHRS4-003, DHRS4-AS1-004, DHRS4-204, DHRS4-004, DHRS4-AS1-003, DHRS4-AS1-005, DHRS4-AS1-002, DHRS4-AS1-007, DHRS4-005, DHRS4-007, DHRS4-006
**chr1:62660188-62660861**	7	4.67E-07	-3.02E-02	L1TD1-001
**chr6:151646312-151646957**	9	1.07E-03	4.81E-02	AKAP12-002

[a] - Adjusted with Bonferroni correction

Table S8: DMR results for DMRcate meta-analysis BDI-II threshold-20 variable

DMRs	# CpGs	P-Value[a]	Max Effect	Overlapping Promoters
**chr10:70321874-70322442**	3	1.50E-02	3.68E-02	TET1-001
**chr7:155174991-155175340**	3	1.57E-02	3.81E-02	AC008060.7-001
**chr18:67069959-67070461**	6	1.78E-06	-2.35E-02	DOK6-001
**chr5:1393934-1394633**	7	6.93E-08	4.17E-02	NA
**chr8:70378380-70378994**	7	3.28E-04	3.39E-02	SULF1-201, SULF1-001, SULF1-008, SULF1-009, SULF1-010
**chr2:27530670-27531535**	10	3.81E-06	-2.39E-02	UCN-001
**chr8:1764878-1765820**	12	1.06E-04	-1.26E-02	MIR596-201
**chr6:33047944-33048879**	15	2.64E-06	4.56E-02	HLA-DPB1-002, HLA-DPA1-004, HLA-DPA1-001, HLA-DPB1-008, RPL32P1-001, HLA-DPA1-002, HLA-DPB1-006, HLA-DPA1-005, HLA-DPB1-009, HLA-DPB1-005, HLA-DPB1-007

[a] - Adjusted with Bonferroni correction

Table S9: Results for cg08667740 and cg22868225 for this study and the Viuff, A et al. study

	Drakenstein Child Health Study[a]		Viuff, A. et al. ALSPAC mid-pregnancy depression[b]		Viuff, A. et al. Generation R Study[c]	
CpG sites	Effect	P-value[d]	Effect	P-value	Effect	P-value
cg08667740	-1.69E-06	0.944	-0.025	3.90E-08	0.003	0.186
cg22868225	-6.17E-05	0.780	-0.005	5.98E-08	-0.001	0.672

a - in association with the EPDS 13 threshold depression variable
b - in association with the EPDS 12 threshold depression variable
c - in association with the Brief Symptom Inventory (BSI) 0.80 threshold variable
d - adjusted with Bacon and Cate

Table S10: Results for cg06808585, cg05245515, and cg15264806 for this study and the Cardenas, A et al. study

	Drakenstein Child Health Study[a]		Cardenas, A. et al. Discover cohort Project[a]		Cardenas, A. et al. Generation R Study[b]	
CpG sites	Effect	P-value[c]	Effect	P-value[d]	Effect	P-value
cg06808585	-3.64E-03	0.398	3.10	<0.05	0.04	0.96
cg05245515	5.82E-03	0.167	-1.59	<0.05	0.28	0.29
cg15264806	-1.18E-04	0.567	0.14	<0.05	0.05	0.63

a - in association with the EPDS 13 threshold depression variable
b - in association with the Brief Symptom Inventory (BSI) 0.80 threshold variable
c - adjusted with Bacon and Cate
d - FDR

Table S11: Correlation between brain and blood DNAm for CpG sites and DMRs
[a] - These values came from IMAGE-CpG

DMR	CpG sites	DNAm Correlation Across Brain and Blood[a]	P-value[a]
N/A	cg04859497	-0.184	0.422
chr8:70378380-70378994	cg15351186	0.194	0.399
	**cg07051728**	**0.499**	**0.023**
	cg12181083	0.166	0.470
	cg04845579	0.216	0.346
	cg02283643	0.182	0.428
	cg07073960	0.201	0.380
	cg00613562	-0.022	0.926
chr18:67069959-67070461	**cg03790988**	**0.439**	**0.048**
	cg20103519	0.160	0.487
	cg19513940	0.079	0.733
	cg00433861	-0.177	0.442
	cg13890379	-0.236	0.301
	cg20169576	0.079	0.733
